# Membrane disruption attenuates agonist potency in prostanoid receptors

**DOI:** 10.1042/BCJ20253332

**Published:** 2025-10-17

**Authors:** Uurtuya Hochban, Imke Wallenstein, Michaela Ulrich, Alwina Bittner, Lisa Spänig, Katharina Klingelhöfer, Sebastian Neumann, Torsten Steinmetzer, Moritz Bünemann, Michael Kurz

**Affiliations:** 1 Department of Pharmacy, Marburg University, Institute of Pharmacology and Clinical Pharmacy, Marburg, Germany; 2 Department of Pharmacy, Marburg University, Institute of Pharmaceutical Chemistry, Marburg, Germany

**Keywords:** Förster resonance energy transfer (FRET), G protein‐coupled receptor (GPCR), microscopy, molecular cell biology, prostaglandin, saponin membrane disruption

## Abstract

G protein-coupled receptors (GPCRs) are key signal transducers and the target of about one-third of all FDA-approved drugs. Many structural and pharmacological studies rely on disrupted membrane conditions, such as purified receptors in artificial systems or radioligand binding assays using membrane fragments, even though it had not been systematically studied whether membrane integrity affects GPCR function. To address this, we developed Förster resonance energy transfer (FRET)-based GPCR conformation sensors to directly measure receptor activation in both intact and disrupted membranes. Our results show that while some GPCRs remain unaffected, prostanoid receptor conformation sensors exhibit a strong dependence on membrane integrity: their agonist and antagonist potencies decrease up to 30-fold upon membrane disruption, revealing a crucial role of the membrane integrity in ligand–receptor affinity. Validation with wildtype receptors in functional signaling assays confirmed that these effects reflect genuine receptor characteristics rather than unspecific signals from the sensor design. We ruled out several factors that could explain the loss of affinity but were unable to fully elucidate the mechanism behind this phenomenon. Nevertheless, this effect may introduce bias into structural and pharmacological studies. It is therefore essential to account for membrane integrity and to employ optimized experimental strategies to ensure robust and reliable data interpretation.

## Introduction

G protein-coupled receptors (GPCRs) constitute the largest superfamily of membrane proteins and play an important role in many physiological processes such as blood pressure regulation or analgesia [1–3]. Activated by various extracellular stimuli, GPCRs transmit signals into the cell [4]. Upon agonist binding, the receptor can interact with G proteins, arrestins, or other effectors [5–7]. Numerous factors influence GPCR function, many of them depend on the host cell and the specific membrane microenvironment. Cell-specific properties such as the composition of the plasma membrane, including the amount of cholesterol and the abundance of various phospholipids, as well as the membrane potential, also have an influence on receptor activity [8–11]. Additionally, ions play an important role in GPCR function, with sodium ions acting as well-established negative allosteric modulators of class A GPCRs [12].

In contrast, it is not yet well understood whether membrane integrity influences GPCR function. Membrane integrity here refers to the intact lipid bilayer that preserves the cellular milieu and provides the native environment for membrane proteins. GPCRs are frequently studied in disrupted membrane environments, including purified receptors in artificial systems for structural studies, receptors in membrane fragments for radioligand binding assays, and receptors in permeabilized cells for G protein coupling studies [13–16]. However, the potential impact of membrane disruption on receptor properties remains insufficiently characterized.

To address this gap, we employed ratiometric Förster resonance energy transfer (FRET)-based GPCR conformation sensors, which allow real-time monitoring of receptor activation in both intact and non-intact cells. These conformation sensors are a well-established tool to study direct effects of ligands on GPCR activity and to investigate their dynamics and intrinsic properties [17–19]. To compare the activity of different GPCRs both in intact and non-intact cells, we developed several FRET-based GPCR conformation sensors in addition to those that have been published previously [11,18–21]. Based on initial findings showing a strong effect of membrane disruption on the prostaglandin E receptor subtype 4 (EP_4_ receptor), we focused on the family of prostanoid receptors and compared them with a number of non-prostanoid GPCRs such as the free fatty acid receptor 3 (FFA3 receptor), adrenoceptor alpha 2A (α_2A_-adrenoceptor), endothelin receptor type B (ET_B_ receptor), and the sphingosine-1-phosphate receptor 3 (S1P_3_ receptor). Our findings revealed that while some GPCRs remain largely unaffected by membrane disruption, others—particularly members of the prostanoid receptor family—exhibit a strong dependence on membrane integrity. Notably, we observed that agonist and antagonist potencies for prostanoid receptors decreased upon membrane disruption, suggesting that the native membrane environment plays a crucial role in ligand binding. Although we were able to rule out several potential contributing factors, the precise mechanism underlying this sensitivity remains unresolved.

## Results

### Loss of agonist potency in permeabilized cells or membrane fragments observed for several prostanoid receptor conformation sensors

**Figure 1:**
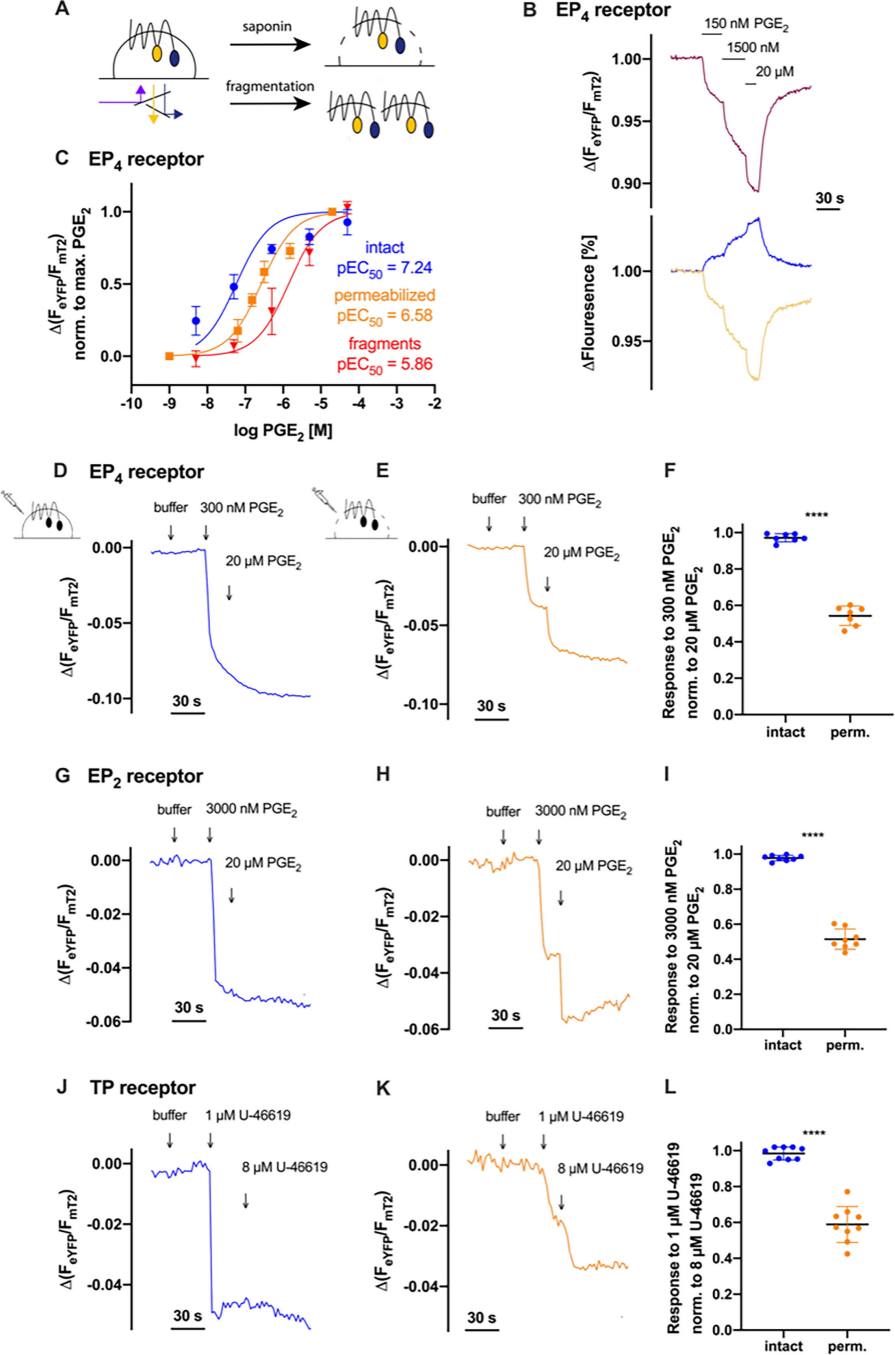
Loss of agonist potency in permeabilized cells or membrane fragments observed for several prostanoid receptor conformation sensors. (**A-L**) Receptor activity was measured using FRET receptor conformation sensors stably expressed in HEK293 cells. (**A**) Schematic overview of the conditions used for FRET measurements: intact cells (left), saponin-permeabilized cells (top right), and membrane fragments (bottom right). Saponin-permeabilized cells were measured in single-cell FRET recordings using a pressurized perfusion system containing either an internal buffer mimicking the cytosolic ionic composition or the same buffer supplemented with the indicated concentration of agonist. The intact cells and membrane fragments were measured in FRET recordings on a plate reader. (**B**) Representative trace (out of *n* = 7) of saponin-permeabilized cells expressing the EP_4_ receptor conformation sensor (top) with corresponding mT2 (cyan) and eYFP (yellow) emission traces (bottom). The emission ratio of eYFP/mT2 was corrected for photobleaching and plotted. (**C**) Concentration–response curves of the EP_4_ receptor conformation sensor measured in intact (blue, *n* = 3, pEC_50_: 7.24), saponin-permeabilized cells (orange, *n* = 7, pEC_50_: 6.58), and in membrane fragments (red, *n* = 3, pEC_50_: 5.86). Measurements in intact cells and membrane fragments were performed side by side. (**D-K**) Representative traces of single-cell measurements of the (**D-F**) EP_4_, (**G-I**) EP_2_, or (**J-L**) TP receptor conformation sensors with Δ(F_eYFP_/F_mT2_) over time in (**D, G, J**) intact (blue) or (**E, H, K**) saponin-permeabilized (orange) cells using a pipetting protocol. The cells were tested with a final concentration of 300 nM PGE_2_, 3000 nM PGE_2_, or 1 µM U-46619, respectively, which equals about ten times EC_50_ of the respective sensors present in intact cells, followed by a saturating concentration of 20 µM PGE_2_ or 8 µM U-46619. (**F, I, L**) Those responses of about 10 times EC_50_ were then normalized to the response of the saturating agonist concentration within the same cell and plotted. The normalized response of approximately ten times EC_50_ was significantly different between the two groups for all three receptor sensors (EP_4_ receptor sensor: unpaired *t* test with Welch’s correction: ****P<0.0001, each *n* = 7; EP_2_ receptor sensor: unpaired *t* test with Welch’s correction: ****P<0.0001, each *n* = 8; TP receptor sensor: unpaired *t* test with Welch’s correction: ****P<0.0001, each *n* = 9). Data are shown as mean ± SD with *n* = 7–9 for single-cell measurements or *n* = 3 for multiple-cell measurements.

To address whether membrane integrity influences GPCR activity, we utilized the advantages of FRET-based GPCR conformation sensors, which allow direct, real-time measurement of receptor dynamics. Importantly, the conformation sensors employed here retain the binding properties of their native GPCR counterparts, as demonstrated previously [18,19], and since no signal amplification is involved, the FRET signal change is considered to represent a direct, linear measurement of agonist–receptor interaction. Our GPCR conformation sensors share the same overall architecture with eYFP as acceptor within the third intracellular loop and mT2 as the donor in the C-terminus analogous to the TP receptor conformation sensor [11]. The C-terminal region of the GPCR was mostly truncated to restrict the flexibility of the fluorescent protein mT2. Agonist binding leads to a concentration-dependent conformational change within the receptor. The outward movement of helix 6, typical for class A GPCRs [22], increases the distance between donor and acceptor, leading to reduced FRET efficiency and thus a decreasing emission ratio, as exemplified by the EP_4_ receptor conformation sensor (Figure 1A–B) [18]. To study the influence of membrane integrity, we compared prostaglandin (PG) E_2_-induced alterations in FRET in single-cell measurements of HEK293 cells stably expressing the EP_4_ receptor conformation sensor under both intact and saponin-permeabilized conditions (Figure 1C). Since all of our FRET conformation sensors are ratiometric, the readout reflects relative changes in donor/acceptor emission and is therefore independent of expression levels or absolute fluorescence intensity. We observed a right shift of the concentration-response curve after the loss of membrane integrity compared with the same measurements performed in intact cells. To rule out detergent effects as the cause for the rightward shift, we conducted experiments with membrane fragments. These membrane fragments were generated by snap-freezing with liquid nitrogen, again showing a strong potency loss (Figure 1C). To circumvent perfusion issues caused by the lipophilic nature of the agonist and to increase the throughput, we established a simplified pipetting protocol (Figure 1D–E, G–H and J–K). A defined volume of internal buffer was placed into the measurement chamber. In the course of each measurement, a buffer solution was first added, followed by a test concentration of about 10 times EC_50_ in intact cells and a saturating reference concentration of the same agonist. For each trace, the response to the test concentration was divided by the reference concentration and the obtained ratio was plotted (Figure 1F, I and L). This protocol also enabled the analysis of other prostanoid receptors. Therefore, experiments were conducted with HEK293 cells stably expressing the EP_4_ receptor, prostaglandin E receptor subtype 2 (EP_2_ receptor) or thromboxane A_2_ receptor (TP receptor) conformation sensors. The EP_2_ receptor conformation sensor constructed in this work was also interesting as it shares PGE_2_ as endogenous agonist with the EP_4_ receptor. A concentration–response curve for this construct yielded a pEC_50_ value of 6.68 for PGE_2_ (Supplementary Figure S1), which is right-shifted by approximately one order of magnitude compared with the EP_4_ receptor, aligning well with previous literature [13]. The stably expressed TP receptor conformation sensor used for our measurements has already been described [11]. We worked with U-46619, a stable analog of PGH_2_, to activate the TP receptor. In agreement with the observations of single-cell measurements using a pressurized perfusion system or multiple-cell measurements in microplates for the EP_4_ receptor, we observed for all three conformation sensors a full response to the test concentration in intact cells while saponin-permeabilized cells showed a substantial loss in potency. The EP_4_ receptor conformation sensor measured in saponin-permeabilized cells showed a response of 56% ± 1% (Figure 1E–F) to the same test concentration inducing a full response in intact cells, the EP_2_ receptor a response of about 51% ± 6% (Figure 1H–I), and the TP receptor a response of 59% ± 1% (Figure 1K–L). The responses are normalized to a saturating concentration within the same cell.

### Potency loss of prostanoid receptors in non-intact cells is not a universal feature of GPCR conformation sensors

**Figure 2:**
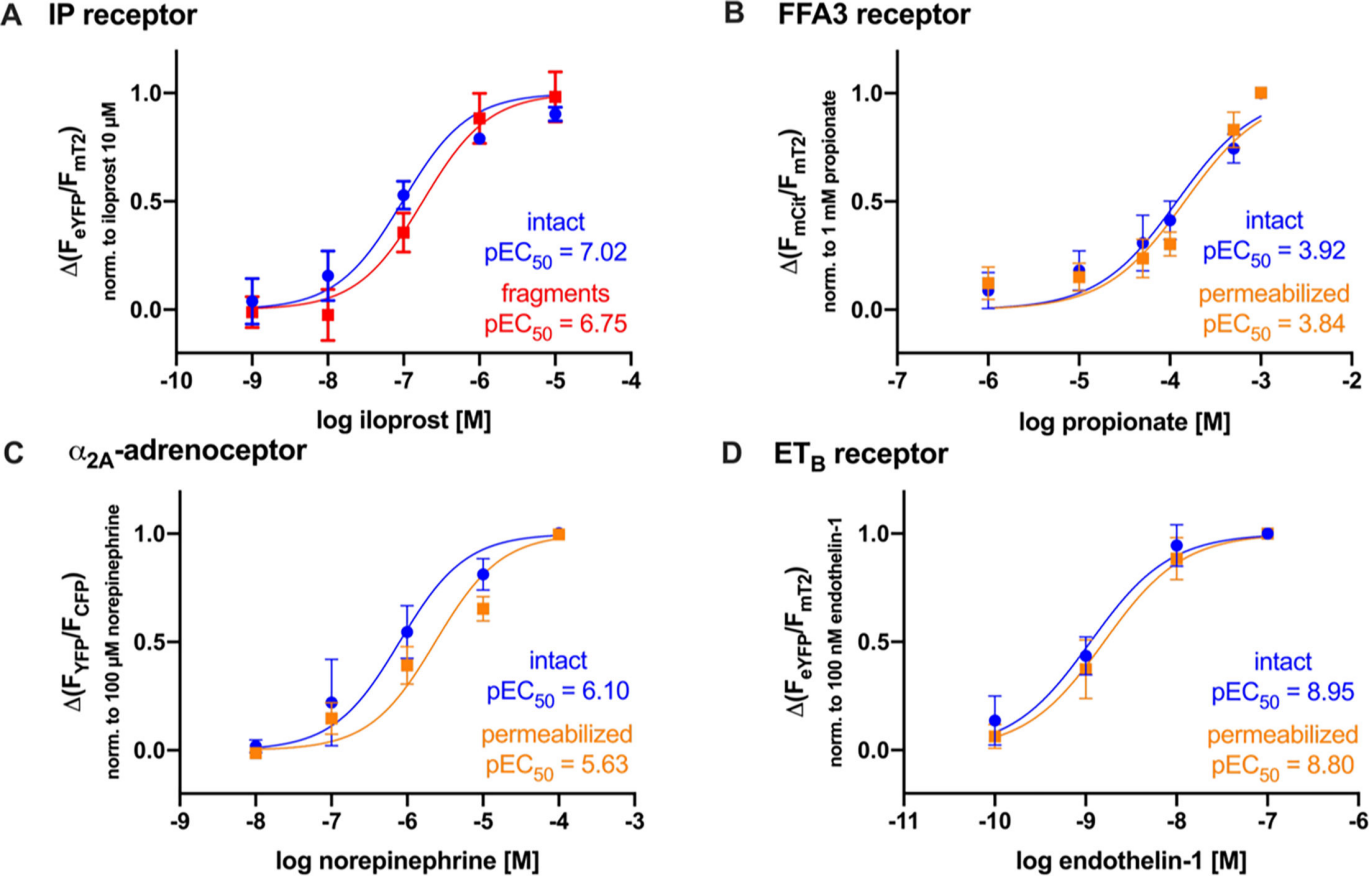
Potency loss of prostanoid receptors in non-intact cells is not a universal feature of GPCR conformation sensors. (**A-D**) FRET measurements of either intact (blue) or saponin-permeabilized (orange) or membrane fragments (red) of HEK293 cells stably expressing the (**A**) IP receptor, (**B**) FFA3 receptor, (**C**) α_2A_-AR CAM, or (**D**) ET_B_ receptor conformation sensors. (**A**) shows the concentration–response curve of the IP receptor conformation sensor measured in intact cells (blue) or membrane fragments (red) in multiple-cell measurements on a plate reader. In (**B-D**), single-cells were measured using a pressurized perfusion system applying increasing concentrations of either (**B**) propionic acid (*n* = 10–11 per condition per data point), (**C**) norepinephrine (*n* = 11 per condition per data point), or (**D**) endothelin-1 (*n* = 8 per condition per data point) and normalized to the response evoked by a saturating concentration applied within the same cell. Data are shown as mean ± SD with *n* = 8–11 for single-cell measurements or *n* = 6 for multiple-cell measurements.

Next, we investigated other FRET-based conformation sensors to find out whether the observed loss of potency was exclusive for the EP_4_, EP_2_, and TP receptor conformation sensors. We therefore focused on other prostanoid receptors such as the prostaglandin I_2_ receptor (IP receptor) and prostaglandin D_2_ receptor 2 (DP_2_ receptor) but also on non-prostanoid receptors such as the FFA3 receptor, α_2A_-AR [19], and ET_B_ receptor, examining the potencies of their endogenous agonists or stable analogs in saponin-permeabilized cells or membrane fragments. The IP and DP_2_ receptor conformation sensors were investigated in multiple-cell FRET measurements in intact cells or membrane fragments. For this purpose, we generated a FRET-based conformation sensor for the IP receptor and established a corresponding stable cell line. The obtained concentration–response curve showed a significant right shift of the pEC_50_ value in membrane fragments. This right shift was compared with the EP_4_, EP_2_ , and TP receptor conformation sensors, which were less robust (Figure 2A, S2D). However, the DP_2_ receptor conformation sensor [20] showed almost no response to the agonist PGD_2_ in membrane fragments, prepared from a stable cell line. In contrast, intact cells from the same line, measured side by side under identical conditions, exhibited a strong response to the agonist, highlighting the importance of membrane integrity for the DP_2_ receptor activation (Supplementary Figure S2A-C). The three chosen non-prostanoid receptors, the α_2A_-adrenoceptor (α_2A_-AR), the ET_B_ , and the FFA3 receptors belong to different subfamilies and are distinct but share the common feature of being class A GPCRs, like the prostanoid receptors. Additionally, their endogenous ligands differ significantly from the highly lipophilic prostanoid receptor ligands—propionate is a charged molecule, norepinephrine a catecholamine, and endothelin-1 a peptide. To investigate the influence of the membrane integrity on the FFA3 and ET_B_ receptors, we constructed FFA3 and ET_B_ receptor conformation sensors. For the construction of the FFA3 receptor conformation sensor, we used mCit instead of eYFP as FRET acceptor to minimize artifacts related to pH alterations [23]. With propionate, we were able to measure a concentration-dependent activation of the receptor conformation sensor (Supplementary Figure S3A). Similarly, we activated the ET_B_ receptor conformation sensor with endothelin-1 in intact cells in a concentration-dependent manner (Supplementary Figure S3B). Both conformation sensors showed a robust signal change upon agonist stimulation, and the observed pEC_50_ values were in agreement with the literature [24,25]. To investigate the α_2A_-AR, we used the previously published conformation sensor [19]. Single-cell FRET measurements of saponin-permeabilized HEK293 cells stably expressing either the FFA3 receptor, α_2A_-AR, or the ET_B_ receptor conformation sensor, side by side with intact cells, were performed with increasing agonist concentrations of propionate, norepinephrine, or endothelin-1, respectively (Figure 2B–D). The resulting concentration–response curves of measurements in intact or permeabilized cells of the FFA3 (Figure 2B) and ET_B_ (Figure 2D) receptor conformation sensors showed no shift indicating a loss of potency after permeabilization. The concentration–response curves of the α_2A_-AR showed a rightward shift upon membrane permeabilization also suggesting a potency loss (Figure 2C).

### Persistent potency loss of EP_4_ receptor in permeabilized cells despite buffer, nucleotide, and ligand adjustments, extending to antagonist affinity and wash-out kinetics

**Figure 3:**
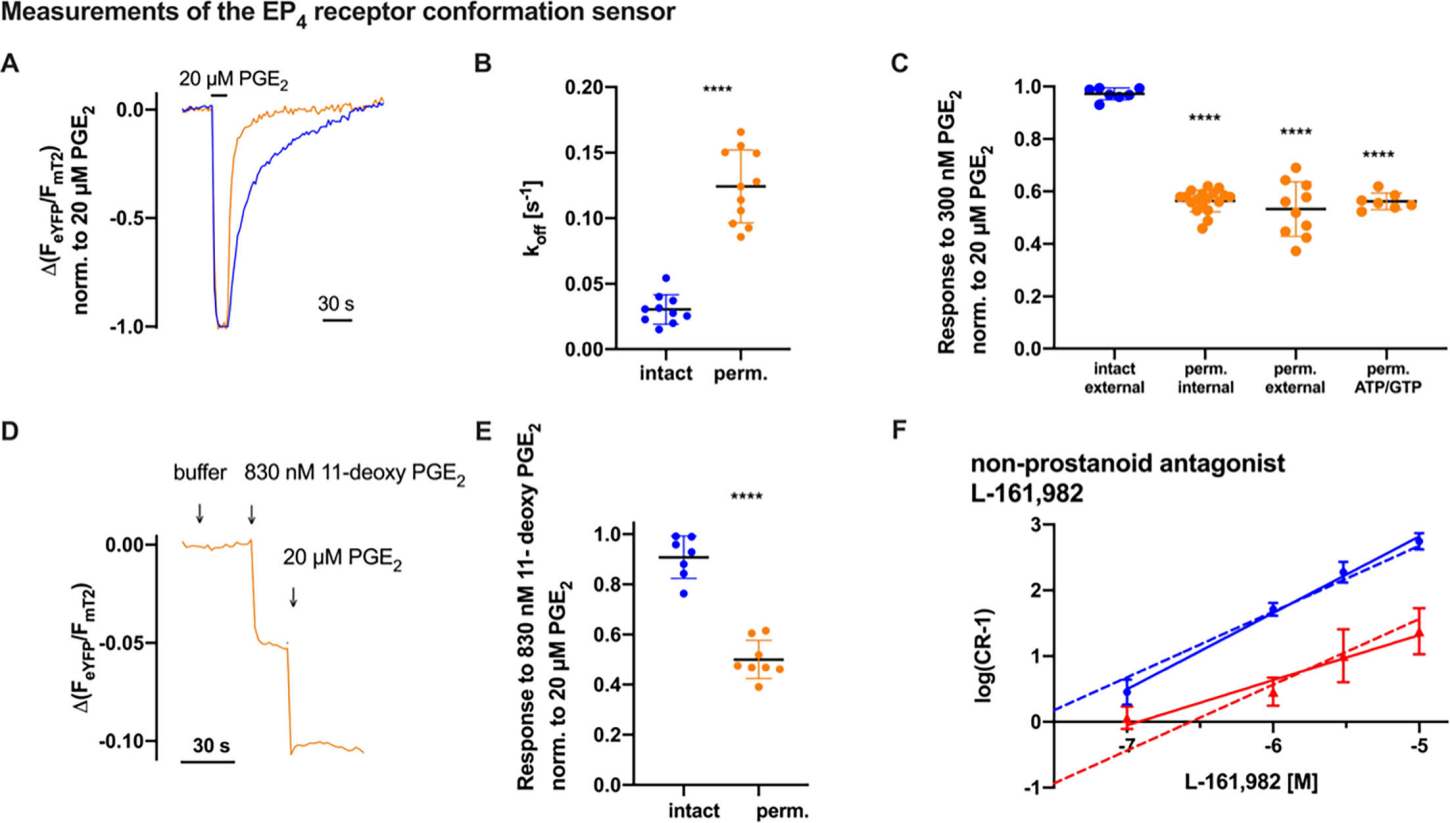
Persistent potency loss of EP_4_ receptor in permeabilized cells despite buffer, nucleotide, and ligand adjustments, extending to antagonist affinity and wash-out kinetics. (**A-F**) FRET measurements of HEK293 cells stably expressing the EP_4_ receptor conformation sensor. (**A**) Single-cell measurements using a pressurized perfusion system with EP_4_ receptor conformation sensor example traces of receptor deactivation wash-out kinetics (blue: intact cells [half-life of example trace: 22 s] or orange: saponin-permeabilized cells [half-life of example trace 4 s]). (**B**) Wash-out kinetics of intact (blue) or permeabilized (orange) cells are fitted to a monoexponential function and the k_off_ is plotted. The two groups (intact: k_off_ 0.03 ± 0.01 s^-1^, *n* = 10; perm.: k_off_ 0.12 ± 0.03 s^-1^, *n* = 11) are statistically significant (unpaired *t* test with Welch’s correction: ****P<0.0001). In (**C**), the cells were measured using a pipetting protocol either intact with external buffer (extracellular ionic composition, *n* = 7), or permeabilized in internal buffer (intracellular ionic composition, *n* = 18), permeabilized in external buffer (extracellular ionic composition, *n* = 10) or permeabilized in internal buffer with ATP/GTP added (intracellular ionic composition with additional 5 mM ATP and 25 µM GTP, *n* = 7), respectively. For each trace, the response to the test concentration was normalized to the response of the reference concentration 20 µM PGE_2_. The obtained ratio was then plotted (blue: intact and orange: permeabilized cells). Statistical analysis using one-way ANOVA followed by Dunnett’s multiple comparisons test revealed a significant difference between all conditions of permeabilized measurements compared with intact measurements. (**D**) Example trace of a measurement of 11-deoxy PGE_2_ in a permeabilized cell with Δ(F_eYFP_/F_mT2_) over time. (**E**) The response of about 10 times EC_50_ of 11-deoxy PGE_2_ in intact (blue) or saponin-permeabilized (orange) cells were then normalized to the response of a saturating PGE_2_ concentration within the same cell and plotted. Statistical analysis using an unpaired t test shows a significant difference between intact (blue) and permeabilized (orange) cells (****P<0.0001, *n* = 7 [intact], *n* = 8 [perm.]). (**F**) Double log plot of PGE_2_-activated EP_4_ receptor conformation sensor (CR-1)-values dependent on the concentration of the non-prostanoid antagonist L-161,982 in intact cells (blue; pK_B_ [abscissa intercept]: 7.7) or membrane fragments (red; pK_B_ [abscissa intercept]: 6.6), see Methods section for calculation. Solid lines show the fitted linear regression (slope value intact cells [blue]: 1.16 [95% CI: 0.96 to 1.37] or membrane fragments [red]: 0.69 [95% CI: 0.12 to 1.18]) and dashed lines the respective linear regression with a slope constrained to 1. The underlying data of the Schild plot analysis are displayed in Supplementary Figure S5A-D. Data are shown as mean ± SD with *n* = 7–18 for single-cell measurements or *n* = 3 for multiple-cell measurements.

To gain insight into the mechanisms underlying the observed loss of potency, we assessed the contribution of ligand affinity by performing kinetic measurements. Since changes in affinity are expected to affect binding kinetics, a loss in affinity at equilibrium would manifest as either a slower association rate, a faster dissociation rate, or both. Receptor conformation sensors are particularly well suited to directly determine relaxation kinetics after agonist withdrawal [26]. Therefore, we compared relaxation kinetics after agonist wash-out as a readout for receptor–agonist dissociation in intact and permeabilized cells. As a representative of the prostanoid receptor subfamily, the EP_4_ receptor was selected for detailed wash-out kinetic analysis. We performed single-cell FRET measurements using the EP_4_ receptor conformation sensor. To ensure direct comparability, measurements in intact and permeabilized cells were performed side by side under identical conditions, differing only in the permeabilization step. In each experiment, the receptor was stimulated with a saturating concentration of the agonist PGE_2_, followed by buffer-mediated wash-out (Figure 3A). Kinetic data were fitted using a monoexponential function, and the resulting k_off_ values were plotted (Figure 3B). In permeabilized cells, PGE_2_ has roughly four times faster k_off_ kinetics than in intact cells, indicating that reduced ligand-receptor affinity at least partially accounts for the observed potency loss.

Ions are critical modulators of GPCR function, with sodium ions serving as well-established negative allosteric regulators of class A GPCRs [12]. Given their regulatory role, we examined whether changes in ion composition upon membrane permeabilization contribute to the observed potency loss. Intact cells were measured in a saline solution similar to the composition of human plasma, which we refer to as ‘external buffer’ (137 mM NaCl, 5.4 mM KCl, 2 mM CaCl_2_, 1 mM MgCl_2_, 10 mM HEPES, pH 7.3). In contrast, saponin-permeabilized cells were measured using ‘internal buffer’ (100 mM K^+^ aspartate, 30 mM KCl, 10 mM NaCl, 10 mM HEPES, 5 mM EGTA, 1 mM MgCl2, pH 7.3), a buffer solution that mimics the ionic composition of the cytoplasm. The main difference between these buffer solutions is the high concentration of K^+^ and low concentration of Na^+^ in the internal buffer, while it is vice versa in the external buffer. In addition, Ca^2+^ is present in external buffer, whereas it is absent in internal buffer. Therefore, to rule out the ionic composition of the buffer solutions as the cause of the potency loss, we repeated the measurements in saponin-permeabilized cells but replaced internal buffer with external buffer. However, this did not restore the potency for PGE_2_, as the normalized response to 10 times EC_50_ (300 nM PGE_2_) was still 53% ± 3%. Under physiological conditions, the cytosol contains ATP and GTP. Therefore, we performed measurements with internal buffer supplemented with 5 mM ATP and 25 μM GTP [27,28]. With a response of 56% ± 1% to 10 times EC_50_, the absence of ATP or GTP in permeabilized cells did not appear to be the underlying cause of the potency loss (Figure 3C).

Next, we wondered whether the nature of the ligand, PGE_2_ itself, affected the loss of potency. To investigate this question, we acquired 11-deoxy PGE_2_, which differs from PGE_2_ by the absence of a hydroxyl group in position 11 at the ring structure (Supplementary Figure S4A) and performed multiple-cell FRET measurements in intact cells stably expressing the EP_4_ receptor conformation sensor. We obtained a pEC_50_ value for 11-deoxy PGE_2_ of 7.08 (Supplementary Figure S4B-D) and then defined 830 nM 11-deoxy PGE_2_ as test concentration close to saturation. Using our pipetting protocol in single-cell measurements, the response dropped from 91% ± 3% in intact cells to 50% ± 3% in saponin-permeabilized cells (Figure 3D–E), indicating that the ligand itself is not responsible for the observed potency loss.

Last, we analyzed whether membrane disruption-induced potency loss was specific to agonists or a general feature of ligand-receptor interactions extending to antagonist affinity, given that agonists preferentially stabilize the active receptor conformation. To address this, we measured concentration-response curves of the EP_4_ receptor conformation sensor activated by increasing PGE_2_ concentrations in the absence or presence of different fixed concentrations of the non-prostanoid antagonist L-161,982. These measurements were performed side by side in intact cells or membrane fragments using multiple-cell FRET measurements. The resulting pEC_50_ values plotted in a double logarithmic Schild plot revealed a distinct loss of the EP_4_ receptor affinity for the antagonist (Figure 3F, Supplementary Figure S5A-D).

### Potency loss in disrupted membranes as an inherent characteristic of prostanoid receptors

**Figure 4:**
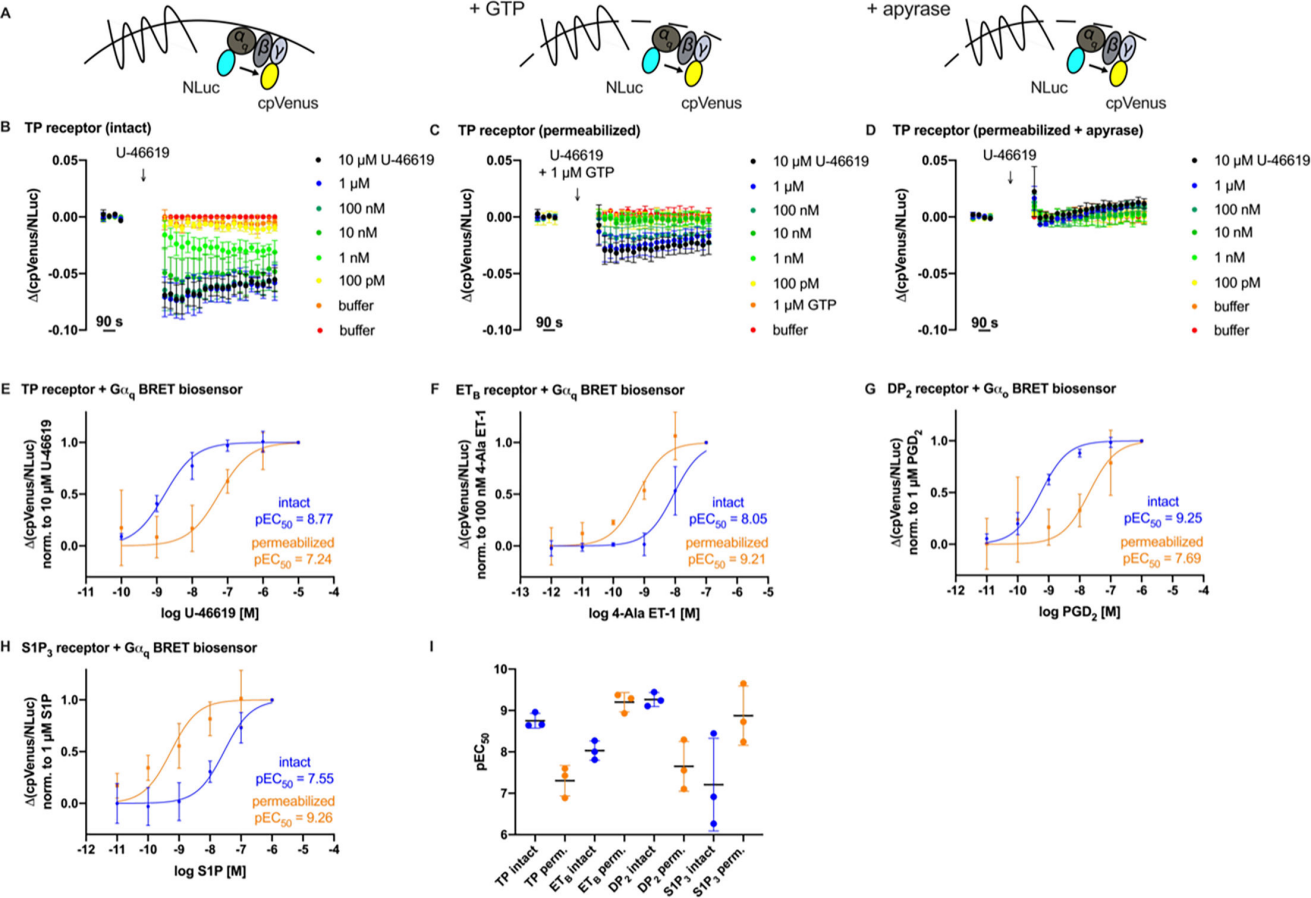
Potency loss of prostanoid receptors in permeabilized cells propagates downstream to the level of G protein activation. G protein activation was measured using BRET-based assays in HEK293T cells transiently co-transfected with the respective wildtype receptors and BRET biosensor constructs. (**A**) Schematic illustration of the experimental setup. G protein activation was assessed under three different conditions: in intact cells (left), in permeabilized cells with added GTP (middle), and in permeabilized cells treated with apyrase (right), as shown in (B–D). (**B-D**) Time courses of BRET signal changes (ΔcpVenus/NLuc) in cells co-expressing the TP receptor and a BRET-based Gα_q_ biosensor. The arrow indicates the time point of ligand application. Measurements were performed in (**B**) intact cells, (**C**) saponin-permeabilized cells with 1 µM GTP, and (**D**) saponin-permeabilized cells treated with 2 U/ml apyrase. (**E**) Corresponding concentration–response curves normalized to a saturating concentration of U-46619 (intact: blue traces or permeabilized: orange traces). (**F-H**) Concentration-response curves of cells co-transfected with (**F**) the ET_B_ receptor and Gα_q_ biosensor, (**G**) the DP_2_ receptor and Gα_o_ biosensor, (**H**) the S1P_3_ receptor and Gα_q_ biosensor. Measurements were performed under the same conditions as shown for the TP receptor (Supplementary Figure S7) and normalized to saturating concentrations of (**F**) 4-Ala endothelin-1 (4-Ala ET-1), (**G**) PGD_2_, or (**H**) Sphingosine-1-phosphate (S1P). In (**I**), all pEC_50_ values of the corresponding concentration-response curves were depicted in one graph. Data show mean ± SD of three independent experiments.

All measurements up to this point were carried out with modified receptors, receptor conformation sensors, in which two fluorophores were introduced into the receptor. Therefore, it was essential to assess the effect of membrane permeabilization on the function of unmodified, wildtype receptors. As the disruption of the plasma membrane is detrimental to many downstream signaling events, we decided to study G protein activation in permeabilized cells by means of BRET. The G protein biosensors contain a small and bright luciferase, NLuc, introduced to the Gα-subunit and N-terminally cpV-tagged Gγ-subunit, as well as a native Gβ-subunit. After adding the substrate of the luciferase, we were able to measure the bioluminescence of the luciferase, allowing resonance energy transfer to the acceptor, cpV, due to the close proximity, when the G proteins are inactive. Upon agonist-induced activation of the receptor and the following activation or dissociation of the G proteins, the BRET emission ratio decreases. However, as the disruption of the plasma membrane leads to a loss of intracellular molecules including GTP, we supplemented GTP [29]. To determine the effective GTP concentration, we designed an experiment in which cells were permeabilized with saponin and exposed to varying GTP concentrations together with the ligand (Supplementary Figure S6). These experiments were conducted in microplates and G protein activation was measured by means of BRET, using G protein biosensors (Figure 4A) [30]. We identified 1 µM GTP as the optimal concentration for G protein activation after permeabilization [28]. As a control, we permeabilized the cells and added apyrase, an enzyme that degrades nucleotides such as GTP, before agonist stimulation. If the cells remained intact, apyrase would not enter, and GTP would still be available, allowing G protein activation. In our case, the absence of any measurable activation following receptor stimulation confirmed the successful permeabilization of the cells. Finally, under these conditions—intact cells, permeabilized cells with 1 µM GTP supplemented or permeabilized cells in the presence of apyrase (Figure 4B–D, Supplementary Figure S7)—we measured the wildtype TP receptor, ET_B_ receptor, and S1P_3_ receptor with Gα_q_ and the DP_2_ receptor with BRET-based Gα_o_ biosensors (Figure 4E–I). Again, we observed the loss of potency after permeabilization only for the prostanoid receptors TP and DP_2_, both of about 30-fold. To our surprise, the absolute BRET change in permeabilized cells for the prostanoid receptors was dramatically lower than in intact cells, indicating the importance of membrane integrity for prostanoid receptors (Supplementary Figure S8). For the ET_B_ receptor and the S1P_3_ receptor, however, we observed an increase in the agonist potency after permeabilization of about 15-fold and 50-fold, respectively (Figure 4F and H, S7A-C, S7G-I, S8B, D, E). This is the opposite effect to that observed in prostanoid receptors. The S1P_3_ receptor also showed lower absolute BRET change amplitudes in permeabilized cells, similar to that observed for the prostanoid receptors (Supplementary Figure S8D). Interestingly, we also observed that within our selection exclusively, the baseline BRET ratio of the DP_2_ receptor was markedly lower in permeabilized cells compared with intact cells (Supplementary Figure S9), consistent with a high degree of basal activity under these conditions. Taken together, these findings confirm that the loss of agonist potency observed for prostanoid receptor conformation sensors upon cell membrane permeabilization also occurs at the level of wildtype receptor-induced signaling.

## Discussion

### Prostanoid receptors lose potency upon membrane disruption due to affinity loss

In the present study, we discovered that the majority of the tested prostanoid receptors exhibit strong dependence on membrane integrity. Membrane disruption reduced the potency of the classic prostanoid receptor family (TP, EP_2_, EP_4_) and the non-classic prostanoid receptor DP_2_, whereas the IP receptor was affected to a lesser extent. In contrast, we could not find any evidence for a major potency loss in GPCRs from other families.

Concentration-response curves of EP_4_ receptor conformation sensors showed a significant rightward shift of the pEC_50_ values upon saponin-permeabilization or when tested against receptors residing in membrane fragments, compared with intact cells (Figure 1C). This loss of potency was confirmed for several prostanoid receptor conformation sensors such as the TP receptor, EP_2_ receptor, and to a lesser extent for the IP receptor (Figures 1 and 2A). Conversely, tested conformation sensors of non-prostanoid GPCRs specifically, the FFA3 receptor, α_2A_-AR, and ET_B_ receptor did not exhibit a major decrease in agonist potency upon disruption of the plasma membrane (Figure 2B–D). These three receptors were chosen based on their representation of different class A GPCR subfamilies, the structural and physicochemical diversity of their agonists and their varying affinities (high, medium, and low). In summary, the major decrease in potency due to permeabilized or fragmented host cells was not a common feature of all GPCR conformation sensors tested in this study but appears to be rather typical for prostanoid receptor conformation sensors. Given that we studied half of the members of the classic prostanoid family (TP, IP, EP_2_, EP_4_) and the non-classic prostanoid receptor DP_2_, we propose that the observed loss of potency after membrane disruption may represent a class effect among prostanoid receptors, highlighting their particular dependence on membrane integrity.

The FRET-based conformation sensors used in this study are known to preserve the ligand-binding characteristics of the corresponding wildtype receptors [18,19]. Since their activation does not involve any amplification steps, the sensor signal is expected to reflect agonist binding in a linear fashion. Therefore, the observed rightward shift in the concentration–response curves upon membrane disruption is best interpreted as a genuine loss of ligand-receptor affinity. Measurements obtained with the EP_4_ receptor conformation sensor - selected as a representative of the prostanoid receptor subfamily—further support this interpretation: faster dissociation kinetics were observed after agonist wash-out (Figure 3A–B) and the association rate also appears to be affected, given that the approx. four-fold acceleration in wash-out kinetics contrasts with the five- to ten-fold EC_50_ shift observed upon saponin-mediated membrane disruption (Figure 1C) [18]. In addition, a comparable decrease in antagonist affinity was identified using Schild plot analysis (Figure 3F, Supplementary Figure S5A-D). Together, these findings strongly suggest that the reduced potency observed upon membrane disruption is primarily due to a loss of ligand-receptor affinity. Classical radioligand binding could in principle provide further insight, but in the case of prostanoid receptors, such experiments are technically extremely demanding. The lipophilic nature of prostaglandins promotes extensive nonspecific membrane binding, and to specifically address the low-affinity state reported by our sensors, binding assays would require GTPγS, which further lowers apparent affinity. Together with the additional affinity loss observed upon membrane disruption, these limitations may render the dynamic range of such assays insufficient. We therefore consider optimized radioligand approaches as valuable but challenging future work rather than part of the present study.

Although these sensors provide a direct and highly informative readout of GPCR activation, they involve receptor modifications - most notably the attachment of large fluorophores—which may, in principle, influence receptor properties. To address this and ensure that the observed effects are not artifacts due to the sensor design, we complemented our findings by studying wildtype receptors. This approach provides a more native receptor environment, albeit with a more indirect readout. Therefore, we refined and applied a previously described G protein activation-based assay to study wildtype receptors in both intact and permeabilized cells [31]. Consistent with the findings at the level of the conformation sensors, the TP receptor, a classical prostanoid receptor, exhibited a strong loss of potency upon permeabilization, whereas the non-prostanoid ET_B_ receptor did not show this effect (Figures 1, 2 and 4E–F). Instead, the ET_B_ receptor displayed an increased affinity after permeabilization likely due to supplemented GTP and enhanced ternary complex stability. Since the applied 1 µM GTP, which was chosen as it allowed robust agonist responses while only causing modest basal activation (Supplementary Figure S6), is certainly below physiological levels [27], we probably enhanced the lifetime or stability of the receptor-G protein interaction as we have described before [26]. As G protein binding allosterically enhances the GPCR affinity for its agonist, this probably explains the increased affinity observed for the ET_B_ receptor. Notably, the same conditions failed to rescue the affinity loss of the TP receptor, highlighting its intrinsic dependence on membrane integrity and suggesting an even greater reduction than observed, given that the ET_B_ receptor gained affinity under 1 µM GTP. Using the same assay, we found that the DP_2_ receptor also reacted with a profound loss of agonist affinity to the disruption of the plasma membrane, whereas the S1P_3_ receptor exhibited a pronounced increase in agonist affinity similar as the ET_B_ receptor (Figure 4G–H). Since the DP_2_ receptor is structurally distinct from classical prostanoid receptors, sharing a low homology and lacking critical amino acids, this suggests that the observed affinity loss extends beyond the traditional prostanoid receptor subfamily [32–34]. An additional observation is the marked reduction of the baseline BRET emission ratio for the DP_2_ receptor in permeabilized cells compared with intact cells (Supplementary Figure S9C). This strongly suggests a high basal activity state of the DP_2_ receptor under these conditions and explains why agonist-induced responses could not be detected in fragmented DP_2_ receptor conformation sensor cells. While this finding does not account for the general affinity loss upon membrane disruption, it represents an intriguing observation in its own right and raises new questions regarding the mechanisms that drive basal activity of the DP_2_ receptor in disrupted membranes.

Taken together, these findings indicate that prostanoid receptors are highly sensitive to membrane integrity and their affinity is drastically reduced when membrane integrity is lost. We would assume that this loss of affinity after membrane disruption—and thus loss of membrane integrity—applies for the entire prostanoid family, as these receptors appear to be particularly dependent on an intact plasma membrane for their proper function.

### Narrowing down the potential causes of prostanoid receptor affinity loss

Understanding the mechanism behind prostanoid receptor affinity loss upon membrane disruption could offer key insights into receptor function and regulation. Receptor affinity can be influenced by various factors such as allosteric modulators, membrane potential, receptor conformation, and the presence of cofactors. Additionally, ligand entry mechanisms and ligand properties could also affect binding affinity. The observed loss of prostanoid receptor affinity upon membrane disruption could arise from any of these factors. Through our experiments, we systematically evaluated these possibilities and were able to exclude several of them, narrowing down the potential mechanisms responsible for this effect. Membrane permeabilization induced by a short exposure to saponin could potentially lead to a detergent-induced alteration in receptor activity and thus influence our single-cell measurements; however, our observations of reduced potency of prostaglandins were reproduced in membrane fragments (Figure 1C), which were generated through snap-freezing without any detergent. Therefore, we can rule out that the loss of potency can be attributed to an effect of detergents.

Ions play a crucial role in GPCR function, with sodium ions acting as well-established negative allosteric modulators of class A GPCRs [12]. Given their regulatory influence, we considered whether changes in ion composition upon membrane permeabilization could contribute to the observed loss of affinity. However, our experiments showed that this effect was independent of electrolyte composition, as variations in sodium and other physiologically relevant ions did not rescue prostanoid receptor affinity. Similarly, the presence or absence of ATP and GTP had no significant impact (Figure 3C). The involvement of albumin as a potential confounder can be excluded, since within each experimental series, buffer conditions were used consistently—either always supplemented with albumin or always without it. Moreover, the observed potency loss was present in both cases: in single-cell FRET experiments of the TP receptor with albumin in the buffer (Figure 1K–L) and in BRET measurements of the TP receptor without albumin (Figure 4E).

In addition, methodological aspects make it unlikely that the observed potency loss originates from changes in the probe microenvironment. Since all of our FRET conformation sensors are ratiometric, the readout reflects relative changes in donor/acceptor emission and is therefore independent of expression levels or absolute fluorescence intensity. Within each experimental series, intact and disrupted cells were always recorded side by side in the identical buffer, and membrane fragments generated by snap-freezing are expected to maintain the lipid bilayer more faithfully than saponin-permeabilized cells, yet the loss of potency was evident in both cases (Figure 1C). Together with the matching results obtained from wildtype receptors using BRET assays (Figure 4), this strongly argues against an artifact of the sensor environment. While we cannot fully exclude contributions from subcellular receptor pools, the PM-dominant distribution under our recording conditions, the persistence of the effect in snap-frozen membrane fragments (Figure 1C), and its reproduction with wildtype receptors in BRET (Figure 4E–H) argue that altered subcellular localization is unlikely to be the primary driver of the potency shift.

One factor that could theoretically account for the observed loss of affinity is the breakdown of the electrical field due to the loss of the membrane potential upon disruption of the cell membrane integrity. Indeed, voltage dependency has been described as a ligand-specific agonist affinity modulator for a number of GPCRs. In contrast with the loss of affinity upon permeabilization or fragmentation, our previous work on the voltage dependence of prostanoid receptors has revealed an increase in affinity for prostaglandins upon depolarization [11]. There is no obvious connection between the voltage dependency of GPCRs and the observed decrease of agonist affinity upon disruption of the cell membrane integrity for prostanoid receptors, and it does not provide a conclusive explanation for the described phenomenon.

Factors such as the physicochemical properties of the ligand and the mechanism of its entry into the binding pocket could also contribute to the observed loss of affinity. A key characteristic of prostanoid receptor function is the entry of the agonist into the orthosteric pocket from the lipid phase of the plasma membrane and not from the extracellular water phase [35]. However, since the S1P_3_ receptor, which shares this ligand entry mode, exhibited an increased affinity upon membrane permeabilization similar with the ET_B_ receptor (Figure 4F and H), this entry pathway is unlikely to be the primary determinant of prostanoid receptor sensitivity to membrane integrity [36,37].

Testing 11-deoxy PGE_2_, a PGE_2_ analog lacking a hydroxyl group, it showed a similar loss of affinity upon membrane disruption, indicating that ligand structure is not the determining factor (Figure 3D–E). This aspect is further supported as conformation sensors of non-prostanoid GPCRs, specifically FFA3, α_2A_-AR, and ET_B_ receptors, did not show significant decrease in affinity upon disruption, despite diverse ligand properties and affinities (Figure 2B–D). Analyzing antagonist binding revealed a similar affinity loss as confirmed by Schild plot analysis (Figure 3F, Supplementary Figure S5A-D). This suggests that membrane disruption broadly affects receptor-ligand interactions through structural or conformational changes.

It is possible that a yet unknown cellular protein or factor could account for the increased affinity of prostanoid receptors in intact cells. While the EP_4_-associated protein has been shown to modulate EP_4_ receptor-mediated immune responses [38], it cannot explain the observed phenomenon, as it does not interact with the EP_2_ receptor. It should also be noted that this factor cannot exert its influence in the present context, as the C-terminus of the EP_4_ receptor—essential for interaction with the EP_4_-associated protein [38]—was truncated in our sensor construct [18]. Furthermore, this factor probably exists across cell types, as EP_4_ receptor affinity for PGE_2_ was similar in MDCK, HEK, and HT22 cells [18]. The identification of a yet undescribed factor that significantly influences the affinity of prostanoid receptors for both endogenous agonists and antagonists is of great interest to the field but lies beyond the scope of this study. It is also important to acknowledge that in permeabilized cells, any protein that is not anchored to the membrane would get lost. Additionally, the receptor conformation sensors are not able to couple effectors like G proteins or arrestins anymore [39].

### Implications for GPCR research and experimental interpretation

These findings may have important implications for GPCR research, particularly for binding studies using disrupted membrane environments for investigations of prostanoid receptors [13,15,16]. Many pharmacological studies assume that GPCR affinity remains unchanged upon membrane disruption, yet our data show that the chosen prostanoid receptors behave differently in intact vs. disrupted membranes. A possible explanation why this affinity loss has not been widely recognized in radioligand binding assays using membrane fragments could be the heterogeneity of experimental conditions. The methods used for affinity determination varied significantly across studies, leading to large variability in retrieved values and limited comparability. Our data suggest that past affinity measurements for prostanoid receptors may need re-evaluation, as ligand binding in fragmented or solubilized receptors may not accurately reflect physiological conditions.

This affinity loss might also be relevant for structural studies, where GPCRs are often examined in purified artificial systems, potentially affecting ligand-binding interpretations. Since similar effects in receptor families beyond prostanoid receptors cannot be ruled out, the impact on G protein binding assays in permeabilized cells also remains unclear but should be considered. The previously unrecognized affinity loss may have led to the use of insufficient ligand concentrations, necessitating a reassessment of past interpretations.

Beyond the immediate impact on affinity measurements, our findings indicate the existence of an unknown molecular factor or mechanism specific to prostanoid receptors that is crucial for their high-affinity ligand binding in intact membranes. The nature of this factor remains unresolved, but its identification could provide key insights into receptor regulation and function. Understanding this mechanism is beyond the scope of this study but represents an important target for future research, as it could reveal novel allosteric modulators, receptor–lipid interactions, or (protein) co-factors involved in prostanoid receptor function.

## Methods

### Plasmids and agonists

cDNAs for the human EP_2_ receptor (prostaglandin E receptor 2, subtype EP_2_ (PTGER2), AY275471, catalog number: #PER0200000), human IP receptor (prostaglandin I2 (prostacyclin) receptor (PTGIR), AY242134, catalog number: #PTGIR000000), human DP_2_ receptor (prostaglandin D_2_ receptor (CRTH2/GPR44), AY507142, catalog number: #CRTH200000) and human S1P_3_ receptor (sphingosine 1-phosphate receptor 3, catalog number: #EDG0300000) were purchased from the Missouri S&T cDNA Resource Center (Bloomsburg, Pennsylvania, U.S.A.). The cDNA encoding for the human TP receptor (here and henceforth referring to the α-isoform) was described previously [40], and the cDNA encoding for the human FFA3 receptor has been kindly provided by Dr. Stefan Offermanns, Max-Planck-Institute for Heart and Lung Research, Bad Nauheim, Germany. The cDNA encoding for the human ET_B_ receptor has been kindly provided from Dr. Michael Schäfer, Rudolf-Boehm-Institute for Pharmacology and Toxicology, University of Leipzig, Leipzig, Germany. The plasmids containing cDNAs of BRET-based G protein biosensors Gα_o1_ (Gβ_3_-T2A-cpVenus-Gγ_9_-IRES-Gα_o1_-NLuc) and Gα_q_ (Gβ_3_-T2A-cpVenus-Gγ_9_-IRES-Gα_q_-NLuc) have been described previously [30]. Already described were the cDNA encoding for the human EP_4_ receptor conformation sensor [18], the human TP receptor conformation sensor [11], human DP_2_ receptor conformation sensor [20], and the murine α_2A_-AR conformation sensor CAM [19].

In this study, we used PGE_2_ (14010), PGD_2_ (12010), U-46619 (16450), L-161,982 (10011565), Iloprost (18215), and 11-deoxy PGE_2_ (14520) (all from Cayman Chemical, Ann Arbor, Michigan, U.S.A.) to investigate prostanoid receptors. All compounds were prepared as stock solutions. PGE_2_ was dissolved in ethanol to prepare a 5 mM stock solution for single-cell FRET experiments, and in dimethyl sulfoxide (DMSO) to prepare a 50 mM stock solution for plate reader measurements. L-161,982 was dissolved in DMSO to prepare a 50 mM stock solution. PGD_2_ and 11-deoxy PGE_2_ were dissolved in ethanol to prepare 50 mM stock solutions. U-46619 was dissolved in ethanol to prepare a 10 mM stock solution. Iloprost was dissolved in ethanol to prepare a 25 mM stock solution. For all FRET experiments involving prostanoid receptors, the stock solutions were further diluted in either internal (100 mM K^+^ aspartate, 30 mM KCl, 10 mM NaCl, 10 mM HEPES, 5 mM EGTA, 1 mM MgCl_2_, pH 7.3) or external buffer (137 mM NaCl, 5.4 mM KCl, 2 mM CaCl_2_, 1 mM MgCl_2_, 10 mM HEPES, pH 7.3) each supplemented with 0.1% bovine serum albumin (bovine serum albumin from heat shock fraction, protease free, fatty acid free, essentially globin free, Sigma-Aldrich, St. Louis, Missouri, U.S.A.). PGD_2_ and U-46619 were also used for BRET measurements on the plate reader; in this case, the stock solutions were diluted only in internal or external buffer without any bovine serum albumin. Sphingosine-1-phosphate (Cat. No.: 1370/1, Tocris, Bio-Techne GmbH, Wiesbaden-Nordenstadt, Germany) was prepared as a 1 mM stock solution in methanol and further diluted in internal or external buffer supplemented with 0.4% bovine serum albumin. Propionic acid (Sigma-Aldrich) was dissolved in internal buffer to prepare a 1 M stock solution. Norepinephrine-bitartrate salt (Sigma-Aldrich) was dissolved in water to prepare a 10 mM stock solution. Endothelin-1 (VWR, Darmstadt, Germany) was dissolved following the manufacturer’s instructions at a final concentration of 100 µM. Unless otherwise stated, these stock solutions were diluted in internal buffer for final use.

### Synthesis of 4-Ala endothelin-1

4-Ala endothelin-1 was used for BRET measurements. The synthesis was carried out according to the following scheme and was described previously [41,42]. Fmoc amino acids, coupling reagents, resins, solvents, and other chemicals for synthesis were purchased from Iris Biotech, Novabiochem, Orpegen, RAPP Polymere, Aldrich, and Acros. Analytical HPLC experiments were performed with a Hitachi Primaide system (column: Nucleodur C_18_, 5 µm, 100 Å, 4.6 × 250 mm, Macherey–Nagel, Düren, Germany) using a linear gradient system with 0.1% TFA in water and 0.1% TFA in acetonitrile as solvents A and B, respectively (20% B – 70% B in 50 min, detection at 220 nm, flow rate 1 ml/min). Preparative purification of 4-Ala-ET-1 was performed with a Knauer Azura HPLC system (column: Nucleodur C_18_, 5 µm, 100 Å, 32 × 250 mm, Macherey–Nagel, Düren, Germany) using the same solvents A and B (linear gradient, 0.5% increase of solvent B per min, flow rate of 20 mL/min, detection at 220 nm). The peptide was obtained as TFA salt after lyophilization. A QTrap 2000 electrospray ionization (ESI) spectrometer (Applied Biosystems) was used for mass determination. Solid phase peptide synthesis was performed on a Syro 2000 peptide synthesizer (MultiSynTech, Witten, Germany) starting with 177 mg of preloaded Fmoc-Trp(Boc)-Wang resin (loading 0.29 mmol/g) in a 2 ml reaction vessel. A standard Fmoc protocol with DMF as solvent was used (double couplings with approximately four-fold excess of Fmoc-amino acid, HOBt, and HBTU, respectively, and 8 eq of DIPEA, 2 × 2 h coupling time). Cleavage of the peptide from resin and deprotection was performed with a mixture of TFA/triisopropylsilane/water (95:2.5:2.5, *v*/*v*/*v*) over 2.5 h at room temperature. After precipitation of the crude peptide in cold diethyl ether, the partially oxidized methionine was reduced by treating the peptide with 0.725 M *N*-methylmercaptoacetamide in water for 72 h at 37°C [43]. Finally, the peptide was purified by preparative reversed-phase HPLC and lyophilized (9.8 mg of white solid TFA salt, purity 97.5% based on detection at 220 nm, analytical HPLC: 24.0 min; ESI-MS: calc. 2366.2, found *m*/*z*: 1184.6 (*M* + 2H)^2+^/2).

### Cloning

### Generation of IP, ET_B_, EP_2_, and FFA3 receptor conformation sensors

The IP, ET_B_, and EP_2_ receptor conformation sensors were generated following a previously described protocol [11]. The fluorescent protein mT2 is flanked by AgeI and EcoRV, and eYFP by NotI and SacII. For amplification, we used Q5 High-Fidelity DNA polymerase (New England Biolabs, Ipswich, Massachusetts, U.S.A.) and afterward NEBuilder HiFi DNA Assembly kit (New England Biolabs) for ligation. For the FFA3 receptor conformation sensor, mCit was used. This fluorescent protein was introduced after amplification with a mutagenesis primer to insert the restriction enzyme sites and digestion with the corresponding restriction enzymes (New England Biolabs) followed by ligation with T4 ligase (New England Biolabs). All primers listed in the following Table 1 were created with SnapGene Viewer (GSL Biotech LLC, San Diego, California, U.S.A).

**Table 1:** conformation sensor cloning primers.

Name	Forward primer (5′→3′)	Reverse primer (5′→3′)
**IP receptor**		
mT2 insertion after G329	CTGTACAAGGATATCAGGAGGGACCCAAGGGC	CACACCGGTCCCTGAGGCGAGCTGG
mT2 as fragment	GGGACCGGTGTGAGCAAGGGCGAGGA	TCCTGATATCCTTGTACAGCTCGTCCATGCC
eYFP insertion after G222	CAAGCCGCGGTCTCTGGGTCCACGGCC	CATAGCGGCCGCGCCCTGGTGGCGCTTC
eYFP as fragment	GCGGCCGCTATGGTGAGCAAGGGCGAG	GACCGCGGCTTGTACAGCTCGTCCATGCC
**ET_B_ receptor**		
mT2 insertion and truncation after L421	TGTACAAGTGAAAGAGGATCCTCTAGAGGGCC	GCTCACTAAGCACGACTGCTTTTCCTCCAA
mT2 as fragment	TCGTGCTTAGTGAGCAAGGGCGAGGAG	GATCCTCTTTCACTTGTACAGCTCGTCCATGCC
eYFP insertion after I309	CCGCGGGCTTTAAATGATCACCTAAAGCAGAGACG	AGCGGCCGCTAAAATCTGCATGCCACTTTTCTTTCTCA
eYFP as fragment	TTTTAGCGGCCGCTATGGTGAGCAAGGGCGAG	TTTAAAGCCCGCGGCTTGT ACAGCTCGTCCATGCC
**EP_2_ receptor**		
mT2 insertion and truncation after Q349	GTAAGATATCCTCGAGTCTAGAGGGCCCG	ACCATACCGGTCTGTGTAGAACAGGAAGTTTGTGTTGC
mT2 as fragment	TACACAGACCGGTATGGTGAGCAAGGGCGAG	CTAGACTCGAGGATATCTTACTTGTACAGCTCGTCCATGCC
eYFP insertion and deletion of amino acids between S237 and G251	AAGCCGCGGGGGGAAAGGGTGTCCATGG	GCGGCCGCCGGAAGGTCCGCAGCGG
eYFP as fragment	GGCGGCCGCATGGTGAGCAAGGGCGAG	CCCCGCGGCTTGTACAGCTCGTCCATGCC
**FFA3 receptor**		
mT2 insertion and truncation after G314	GGGGAGGAGCAGAGAGCG	TCCCTTCTGCTCCTTCAGCTC
mT2 as fragment	AGCTGAAGGAGCAGAAGGGAACCGGTGTGAGCAAGGG	CGCTCTCTGCTCCTCCCCGATATCCTTGTACAGCTCGTCCATGC
Insertion of SacII and HpaI restriction enzyme sites after H217	AGAGGGGGCAGCCACCCGCGGGGGGGGGTTAACCGCCGGCAGAGGAGG	

## Cell culture

In this study, we have used either stably transfected human embryonic kidney 293 (HEK293) cells or transiently transfected human embryonic kidney 293T (HEK293T) cells. The HEK tsA 201 cell line was a kind gift from the Lohse laboratory, University of Würzburg, Germany. All cells were cultured in Dulbecco’s modified Eagle’s medium (DMEM, 4.5 g/l glucose) supplemented with 10% FBS, 2 mM L-glutamine and either 0.4 mg/ml G-418 (stable cell line) or 100 U/ml penicillin and 0.1 mg/ml streptomycin (transient transfection) at 37°C in a humidified atmosphere of 95% air and 5% CO_2_. DMEM, FBS, L-glutamine, penicillin, streptomycin and G-418 sulfate were purchased from Capricorn Scientific GmbH (Ebsdorfergrund, Germany).

### Transfection for FRET measurements

We have generated stable cell lines for all following single-cell FRET measurements on the microscope and multiple-cell FRET measurements in the plate reader. Therefore, HEK293 cells were transfected with 1 µg of the conformation sensor cDNA plasmid with Effectene reagent (Qiagen, Hilden, Germany) according to the manufacturer’s instructions in a dish with 6 cm Ø. Afterward, the cells were cultured under G-418 selection. The day before FRET measurements were performed, the cells were seeded on 25 mm coverslips in six-well plates or microplates. These coverslips or microplates were pre-coated with poly-L-lysine (Sigma-Aldrich).

### Transfection for BRET measurements

Two days before BRET measurements were performed, HEK293T cells were transiently transfected with 3 µg of cDNAs containing TP receptor, ET_B_ receptor, DP_2_ receptor, or S1P_3_ receptor and 3 µg of cDNAs containing Gβ_3_-T2A-cpVenus-Gγ_9_-IRES-Gα_q_-NLuc or Gβ_3_-T2A-cpVenus-Gγ_9_-IRES-Gα_o1_-NLuc using linear 25 kDa PEI reagent (polyethylenimine, PolyScience Inc., Hirschberg an der Bergstraße, Germany). The ratio between DNA and PEI was 1:3, and 50 µl serum-free DMEM was added per 1 µg DNA. The same amount of serum-free DMEM was also added separately to the PEI solution. DNA-DMEM and PEI-DMEM mixtures were incubated for 5 minutes, followed by incubation of the final mixture for another 10 minutes. After counting, the cells were adjusted to 300,000 cells per ml, mixed with the DNA-PEI mixture, and seeded on poly-L-lysine coated microplates.

### Permeabilization

The cells were washed once with external buffer and subsequently incubated 2 minutes for single-cell FRET measurements or 10 minutes for BRET measurements in 0.05% saponin (AppliChem, Darmstadt, Germany) dissolved in internal buffer. Afterward, the cells were washed five times for single-cell FRET measurements or three times for BRET measurements with internal buffer.

### Preparation of membrane fragments

HEK293 cells stably expressing the EP_4_ receptor, IP receptor, or DP_2_ receptor conformation sensor were seeded on 15 cm Ø dishes and grown until optical confluence. The cells were washed once with Dulbecco’s PBS (Capricorn Scientific GmbH), without Ca & Mg and Phenol Red (phosphate-buffered saline, PBS). After adding 5 ml PBS to the cells, the dish was treated with liquid nitrogen until the solution was visibly frozen. These dishes were stored at −80°C until the measurements were carried out. On the day of the measurement, the dishes were removed from the freezer and stored at room temperature until the buffer solution had thawed. The material was collected with a cell scraper and centrifuged (5000 rpm/5 min). The supernatant was discarded, the pellet containing the membrane fragments was resuspended with external buffer containing 0.1% BSA and seeded on microplates.

## Single-cell FRET measurements

The coverslips loaded with stably transfected HEK293 cells containing either the FFA3 receptor, α_2A_-AR CAM, or ET_B_ receptor conformation sensors were clamped in an Attofluor^TM^ cell chamber and were washed once with external buffer. Afterward, they were side by side either permeabilized or directly measured at room temperature by the use of an inverted microscope (Elipse Ti2, Nikon, Düsseldorf, Germany), equipped with a 100× oil immersion objective (Plan Apo λ 100 x/1.45 oil ∞/0.17 WD 0.13, Nikon, Düsseldorf, Germany) and dual excitation and dual emission imaging capabilities. During FRET measurements, mT2 was excited with light of wavelength 435 nm by a LED light source set to an intensity of 20% (pE4000, CoolLED, Andover, U.K.) using an excitation filter (438/24, Semrock, Rochester, New York, U.S.A.) and a dichroic beam splitter (458, Semrock). The fluorescence emission of mT2 and the respective YFP variant was simultaneously collected by a second beamsplitter (488, Chroma, Bellows Falls, Vermont, U.S.A.) and two emission filters (mT2: 474/27, Chroma; YFP: 544/23, Chroma). Fluorescence intensity was measured using a Photometric CMOS Prime 95B Scientific camera (Teledyne Photometrics, Tucson, Arizona, U.S.A.) and the imaging software NIS-Elements Advanced Research 5.21.03 (Nikon) advanced research (Nikon Corporation) and recorded at 10 Hz (FFA3 receptor conformation sensor and α_2A_-AR conformation sensor CAM) or 2 Hz (ET_B_ receptor conformation sensor). For all single-cell FRET experiments, cells were prospectively selected based on a continuous plasma-membrane fluorescence rim and appropriate fluorescence distribution to ensure a PM-dominant receptor signal. During the measurements, the cells were superfused continuously (pressure-driven; VC3-8xP series, ALA Scientific Instruments, Farmingdale, New York, U.S.A.) by either internal buffer or internal buffer containing the indicated agonist. The fluorescence data were corrected for background fluorescence, bleed-through, and false excitation. Then the emission ratio (YFP/mT2) was calculated in Microsoft Excel 2021. Furthermore, the emission ratio was corrected for photobleaching by an exponential baseline subtraction using OriginPro 2016 and normalized to the saturating concentration within the same cell. Further data analysis was carried out using GraphPad Prism 8 (GraphPad Software). The coverslips loaded with stably transfected HEK293 cells containing either the EP_2_ receptor, EP_4_ receptor, or TP receptor conformation sensors were treated and measured analogously to the procedure described for the FFA3 receptor conformation sensor, but on a different setup, which has already been described [44]. The measurements were performed at 0.5 Hz, except for the concentration-response curves of permeabilized cells carrying the EP_4_ receptor conformation sensor or intact cells carrying the EP_2_ receptor conformation sensor, which were measured at 1 Hz. Each individual cell represents one experiment (*n* = 1) and the measurements were carried out on at least two different days at room temperature.

### Multiple-cell FRET measurements

Intact HEK293 cells stably expressing the EP_4_ receptor, IP receptor, or DP_2_ receptor conformation sensors were counted, adjusted to 100,000–120,000 cells per well, and seeded on poly-L-lysine coated microplates one day before measurement. Immediately before starting the measurement, the cells were washed once with external buffer and then 180 µl of external buffer was added to each well.

Membrane fragments of HEK293 cells stably expressing the EP_4_ receptor, IP receptor, or DP_2_ receptor conformation sensors were treated as described in *preparation of membrane fragments,* and 180 µl of the membrane fragment solution was seeded on poly-L-lysine coated microplates. Subsequently, the microplates were centrifuged at 1500 rpm for 3 minutes.

We used a Spark 20M plate reader (Tecan, Männedorf, Switzerland) for multiple-cell FRET measurements of intact cells or membrane fragments. The cells were measured from the bottom and the donor fluorescent protein (mT2) was excited at 430 nm. First, the cells were measured for the baseline emission ratio. After the baseline measurement, 20 µl of internal buffer containing different concentrations of either PGE_2_, Ilo, or PGD_2_ or internal buffer alone were added to the cells. Finally, 20 µl of saturating concentrations of the same agonist or internal buffer alone were added. The emitted donor fluorescence (mT2) and acceptor fluorescence (eYFP) were recorded at 485 nm and 535 nm, respectively. Data were collected using SparkControl^TM^ software. After the measurement, the mT2 and eYFP emissions were exported to Microsoft Excel 2021 and the emission ratio (eYFP / mT2) was calculated. The buffer wells were used as a control and were subtracted. These data were labeled as Δ(F_eYFP_/F_mT2_). The response of each well was normalized to the response to a saturating agonist concentration within the same well. Measurements were performed in at least technical duplicates, with each individual microplate representing one experiment (*n* = 1).

### BRET measurements

Two days before the actual measurement, HEK293T cells were transiently transfected and seeded on microplates. After 48 hours incubation, the cells were washed once with external buffer and, if necessary, permeabilized. At the beginning of the measurement, the final volume per well was 80 µL of either external or internal buffer and 1 µM 8-Benzyl-2-(4-fluorobenzyl)-6-phenylimidazo[1,2 a]pyrazin-3(7*H*)-one (6H-F-Colenterazine) [21] or internal buffer containing 1 µM 6H-F-Colenterazine and 4 U/ml apyrase (Sigma-Aldrich). The measurements were performed using a Spark 20M plate reader (Tecan) at 37°C. Initially, the cells were measured for the baseline emission ratio. After the baseline measurement, 20 µl of external or internal buffer containing differing concentrations of either U-46619, 4-Ala ET-1, PGD,_2_ or S1P or external or internal buffer alone were added to the cells and measured. The agonist solution for permeabilized cells also contained 1 µM GTP (Sigma-Aldrich). The cells were measured from the top and the donor luminescence (NLuc) was recorded at 415 to 470 nm and the acceptor luminescence (cpVenus) at 520 to 590 nm. Data were recorded using SparkControl^TM^ software. After the measurement, the luminescence of NLuc and cpVenus emission was exported to Microsoft Excel 2021. Further evaluation was performed in Microsoft Excel 2021, where the cpVenus/NLuc ratio was calculated and the negative control, where only external or internal buffer was applied, was subtracted. For the saponin-permeabilized measurements with 1 µM GTP, the ratio cpVenus/NLuc was calculated and the response to 1 µM GTP alone was subtracted. The subtracted data were further normalized to the corresponding saturating concentrations. All measurements were performed as duplicates with three independent transfections, while each transfection represents one experiment (*n* = 1). The technical duplicates were averaged, and for the concentration-response curves, the mean of three values from the saturated phase of this average was fitted using GraphPad Prism 8.

### Schild plot

To calculate the concentration-ratio (CR), the pEC_50_ values obtained in the presence of different antagonist concentrations were divided by the pEC_50_ of PGE_2_ alone. The double log plot of (CR-1) values dependent on the concentration of the indicated antagonist was plotted, and a linear regression was fitted with GraphPad Prism 8. To obtain pK_B_ values, the averaged concentration–response curves were fitted with GraphPad Prism 8 to the Gaddum/Schild equation with Schild slope and hillslope constrained to 1, and constrained top and bottom.

### Data analysis and statistics

The data are presented either as single example measurements or as mean ± SD of the stated number of independent experiments. Statistical analysis was performed with GraphPad Prism 8. Differences were considered statistically significant if *P*≤0.05.

Concentration-response curves were fitted in GraphPad Prism 8 using nonlinear regression (*log(agonist) vs. response - variable slope (four parameters*)) with the hillslope set to 1, the bottom constrained to 0 and the top to 1 using the following equation:


$$Y=Bottom+(Top-Bottom)/(1+10^{\left((LogEC50-X)*HillSlope\right)})$$

The ligand wash-out with internal buffer of each single-cell FRET measurement was used to calculate the kinetics by the usage of a nonlinear regression *Dissociation - One phase exponential decay* in GraphPad Prism 8 with Y0 set to 1 and the following equation:


Y=(Y0−NS)∗exp(−K∗X)+NS

## Supplementary material

Online supplementary material 1

## Data Availability

The authors confirm that the data supporting the findings of this study are available within the article and supporting information.

## Open Access

This article has been published open access under our Subscribe to Open programme, made possible through the support of our subscribing institutions, learn more here: https://portlandpress.com/pages/open_access_options_and_prices#conditional

## Abbreviations

4-Ala ET-1: 4 Alanine endothelin 1
Boc: tert-Butoxycarbonyl
CFP: Cyan fluorescent protein
DIPEA: Diisopropylethylamine
DMEM: Dulbecco’s modified Eagle’s medium
DMF: N,N-dimethylformamide
DP2 receptor: Prostaglandin D2 receptor 2
EP2 receptor: Prostaglandin E receptor subtype 2
EP4 receptor: Prostaglandin E receptor subtype 4
ET: Endothelin
ETB receptor: Endothelin receptor type B
FFA3 receptor: Free fatty acid receptor 3
Fmoc: N-(9-fluorenyl)methoxycarbonyl
GPCR: G protein-coupled receptor
HBTU: (2-(1H-benzotriazol-1-yl)-1,1,3,3-tetramethyluronium hexafluorophosphate)
HEK: Human embryonic kidney
HOBt: N-hydroxybenzotriazole
HPLC: High performance liquid chromatography
ICL3: Intracellular loop 3
IP receptor: Prostaglandin I2 receptor
Ilo: Iloprost
MS: Mass spectrometry
NE: Norepinephrine
PG: Prostaglandin
PGD2: (Z)-7-[(1R,2R,5S)-5-hydroxy-2-[(E,3S)-3-hydroxyoct-1-enyl]-3-oxocyclopentyl]hept-5-enoic acid
PGE2: (Z)-7-[(1R,2R,3R)-3-hydroxy-2-[(E,3S)-3-hydroxyoct-1-enyl]-5-oxocyclopentyl]hept-5-enoic acid
S1P3 receptor: Sphingosine-1-phosphate receptor 3
TFA: Trifluoroacetic acid
TP receptor: Thromboxane A2 receptor
U-46619: 9,11-dideoxy-9α,11α-methanoepoxy-prosta-5Z,13E-dien-1-oic acid
YFP: Yellow fluorescent protein
YFP/CFP: Emission ratio
eYFP: Enhanced yellow fluorescent protein
mCit: mCitrine
mT2: mTurquoise 2
mTurquoise fluorescent protein 2: mTurquoise fluorescent protein 2
α2A-AR: Adrenoceptor alpha 2A
