# Supplementary material for: Membrane disruption attenuates agonist potency in prostanoid receptors
Source: Biochem J. 2025 Oct 17;482(20):1565–82. doi: 10.1042/BCJ20253332 (PMC12687449; doi:10.1042/BCJ20253332)
Supplement: Online supplementary material 1 [file bcj-482-20-BCJ20253332-s001.docx]

**Supporting Information**

**of**

**Membrane disruption attenuates agonist potency in prostanoid receptors**

Uurtuya Hochban^1^, Imke Wallenstein^1^, Michaela Ulrich^1^, Alwina Bittner^1^, Lisa Spänig^1^, Katharina Klingelhöfer^1^, Sebastian Neumann^2^, Torsten Steinmetzer^2^, Moritz Bünemann^1^ and Michael Kurz^1^

^1^Marburg University, Department of Pharmacy, Institute of Pharmacology and Clinical Pharmacy

^2^Marburg University, Department of Pharmacy, Institute of Pharmaceutical Chemistry

**Supporting Information**

**
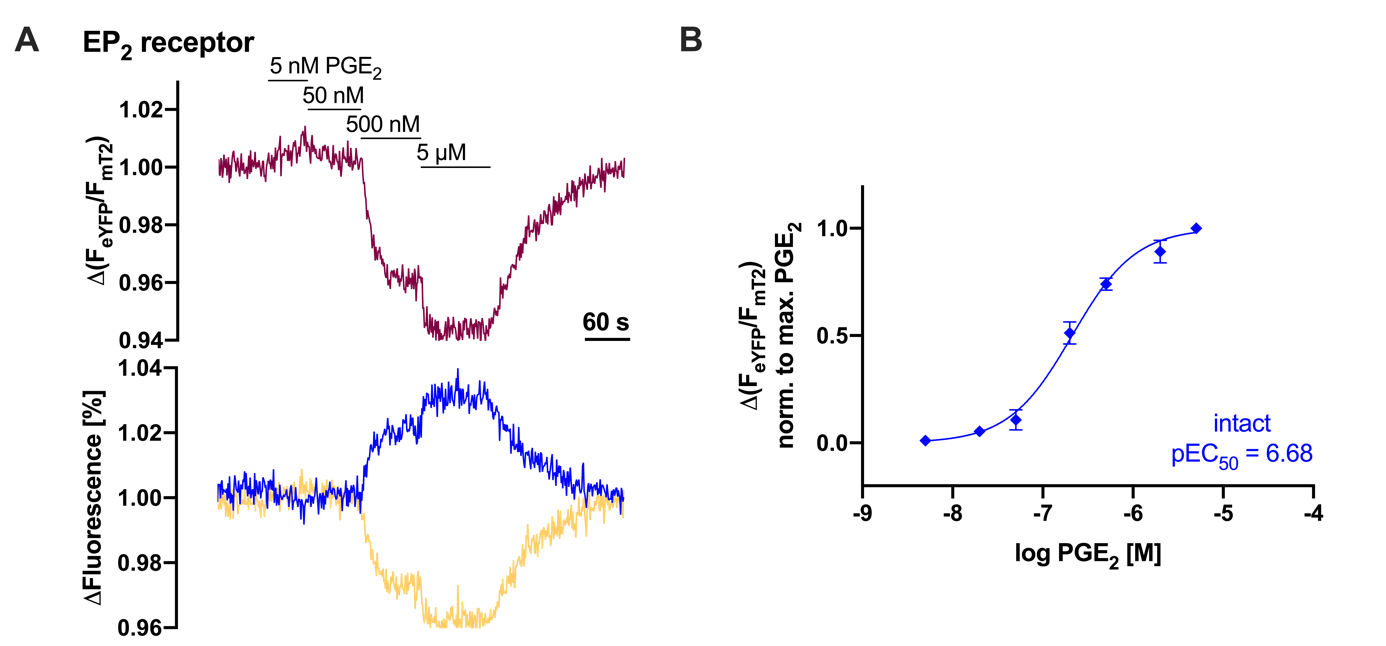
**

**Supp. Figure 1. Single-cell FRET measurements of the EP_2_ receptor conformation sensor activated by PGE_2_.**

(A-B) FRET measurements of HEK293 cells stably expressing the EP_2_ receptor conformation sensor. (A) shows a representative time course of the emission ratio of intact cells treated with external buffer or external buffer containing the indicated amount of PGE_2_ (out of n=5) using a pressurized perfusion system (top) and the corresponding mT2 (cyan) and eYFP (yellow) emission traces (bottom). (B) Concentration–response relationship of EP_2_ receptor conformation sensor activation by PGE_2_ (n=5 per data point). The data for the concentration-response curve was obtained in two separate concentration rows with 20 nM, 200 nM, 2 µM or 5 nM, 50 nM or 500 nM (A) PGE_2_ and normalized to the response of 5 µM PGE_2_ applied within the same cell. Data show mean ± SD.


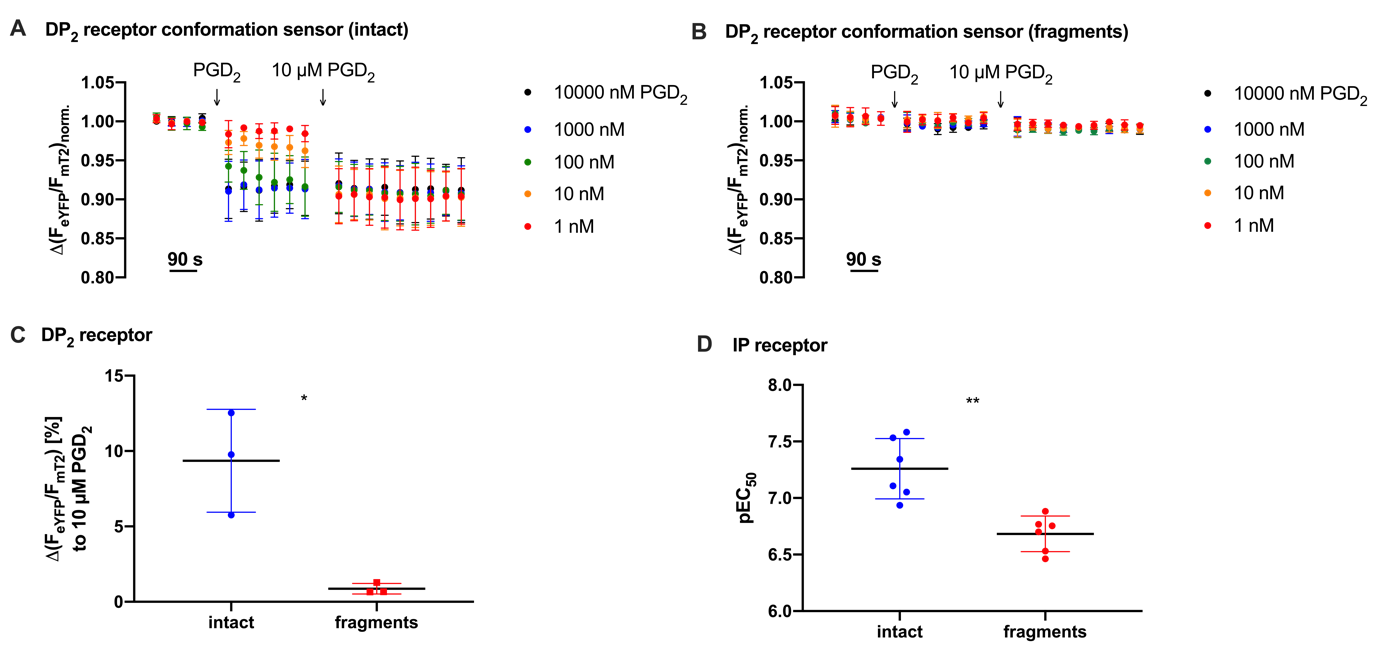


**Supp. Figure 2. Multiple-cell FRET measurements of the DP_2_ and IP receptor conformation sensors in intact cells or membrane fragments.**

(A-B) ΔF_eYFP_/F_mT2_ time courses of stably transfected HEK293 cells expressing the DP_2_ receptor conformation sensor in (A) intact cells or (B) membrane fragments (arrow indicates time point of ligand application). (C) Corresponding E_max_ values of intact cells (blue) or membrane fragments (red); unpaired t test with Welch’s correction: *p=0.0484, each n=3). (D) pEC_50_ values of the IP receptor conformation sensor in intact cells (blue) or membrane fragments (red); unpaired t test: **p=0.0011, each n=6. Data show mean ± SD of three (DP_2_ receptor) or six (IP receptor) independent experiments.


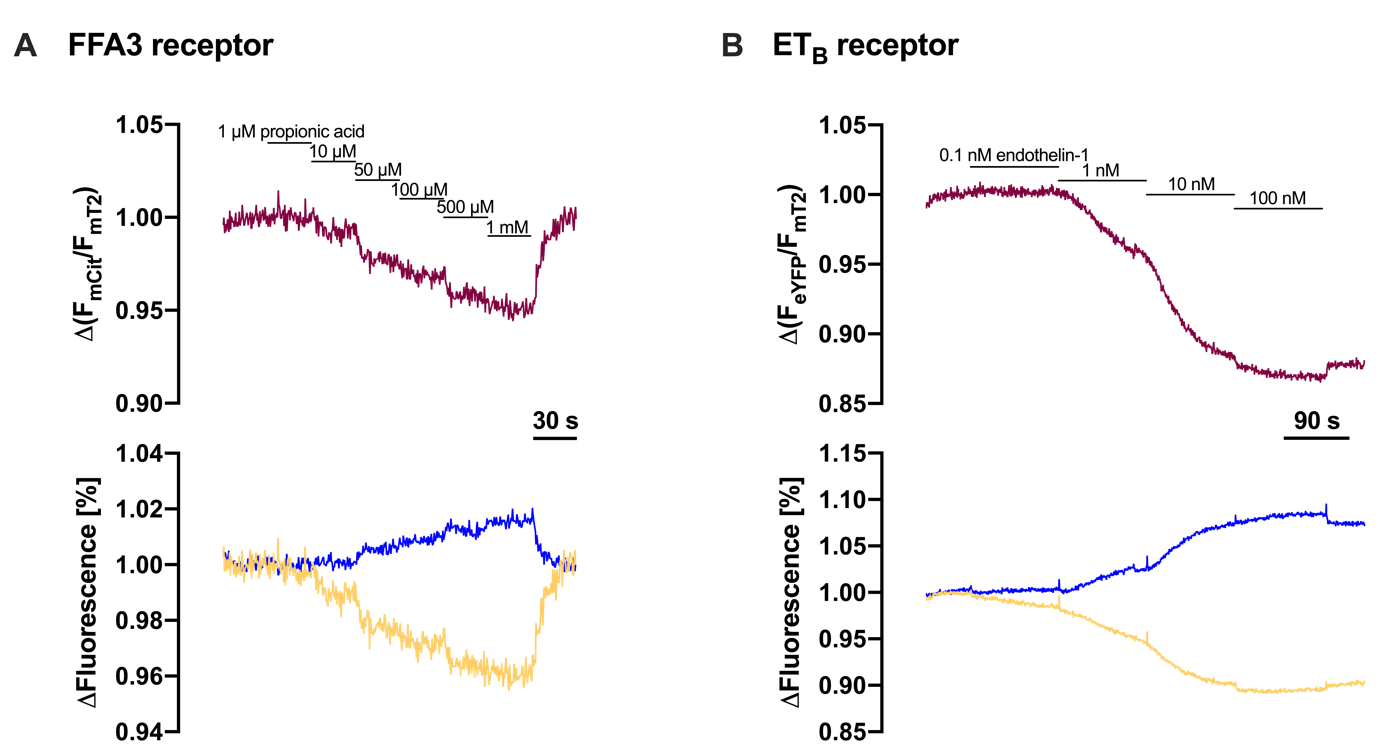


**Supp. Figure 3. Single-cell FRET recordings of the FFA3 receptor and ET_B_ receptor conformation sensors.**

(A-B) Single-cell FRET measurements of HEK293 cells stably expressing the (A) FFA3 receptor or (B) ET_B_ receptor conformation sensor. The measurements were performed in intact cells using a pressurized perfusion system. (A) shows the representative time course of the emission ratio of the FFA3 receptor conformation sensor treated with external buffer or external buffer containing the indicated amount of propionic acid (out of n=11) (top) and the corresponding mT2 (cyan) and mCit (yellow) emission traces (bottom). (B) The representative time course of the emission ratio of the ET_B_ receptor conformation sensor treated with external buffer or external buffer containing the indicated amount of endothelin-1 (out of n=8) (top) and the corresponding mT2 (cyan) and eYFP (yellow) emission traces (bottom).


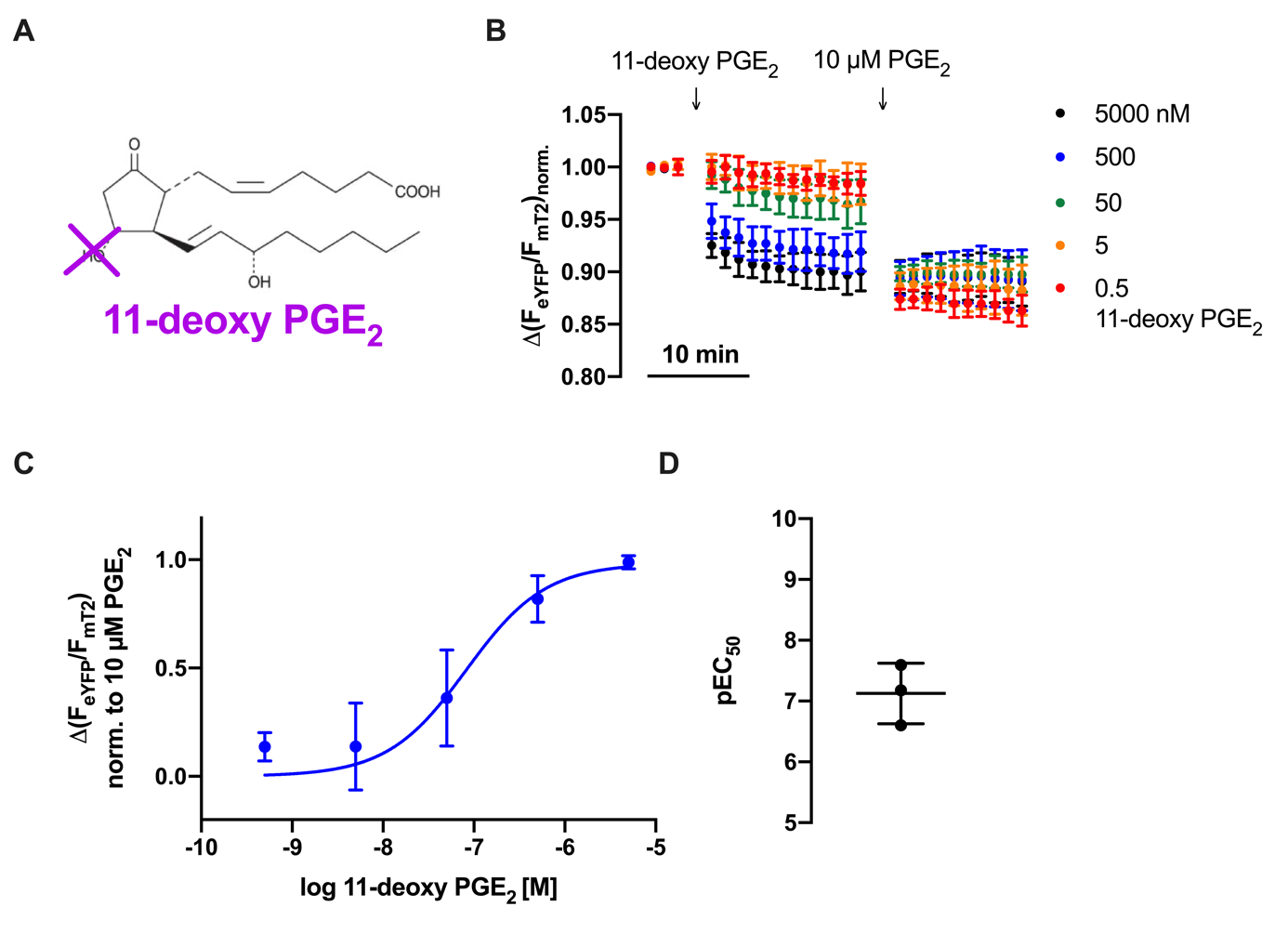


**Supp. Figure 4. Multiple-cell FRET measurements of 11-deoxy PGE_2_ activated EP_4_ receptor conformation sensor in intact cells.**

In (A), the structural formula of PGE_2_ derived from Cayman Chemicals with the OH group crossed out in purple, which is absent in 11-deoxy PGE_2_ showing the difference between the two ligands. (B) ΔF_eYFP_/F_mT2_ time courses of stably transfected HEK293 cells expressing the EP_4_ receptor conformation sensor activated by 11-deoxy PGE_2_ in intact cells (arrow indicates time point of ligand application). Corresponding (C) concentration-response curve and (D) pEC_50_ values (7.08 ± 0.50). Data show mean ± SD of three independent experiments.


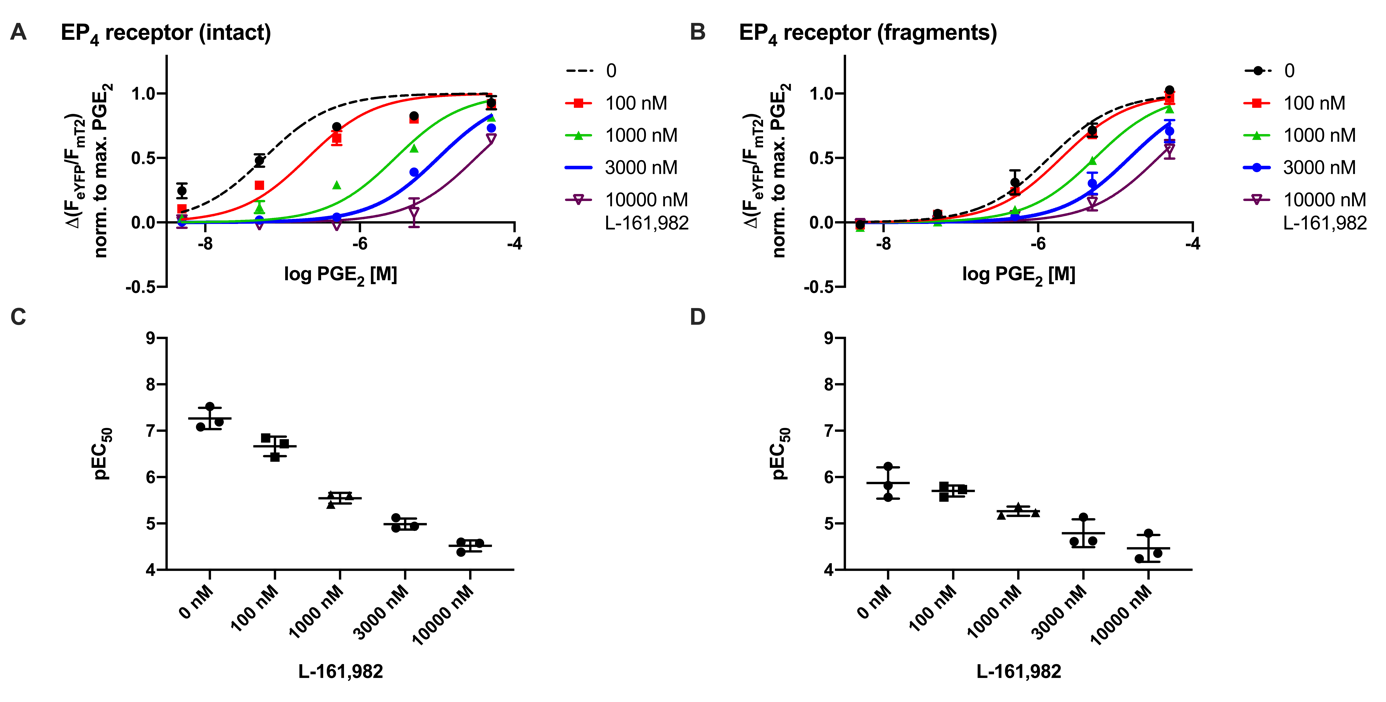


**Supp. Figure 5. Multiple-cell FRET measurements of PGE_2_ activated EP_4_ receptor conformation sensor in intact cells or membrane fragments in absence and presence of L-161,982.**

Underlying data of the Schild plots shown in Fig. 3E. The data for 0 nM L-161,982 has already been shown in Fig. 1C. (A-D) Concentration-response curves of PGE_2_ on the stably transfected EP_4_ receptor conformation sensor measured in the absence or presence of indicated concentrations of the antagonist L-161,982 in (A) intact cells (pEC_50_ PGE_2_: in presence of 0 nM L-161,982: 7.24; 100 nM L-161,982: 6.68; 1000 nM L-161,982: 5.54; 3000 nM L-161,982: 4.99; 10000 nM L-161,982: 4.50) or (B) membrane fragments (pEC_50_ PGE_2_: in presence of 0 nM L-161,982: 5.86; 100 nM L-161,982: 5.70; 1000 nM L-161,982: 5.26; 3000 nM L-161,982: 4.82; 10000 nM L-161,982: 4.45). The corresponding pEC_50_ values of the individual experiments were shown in (C) and (D), respectively.


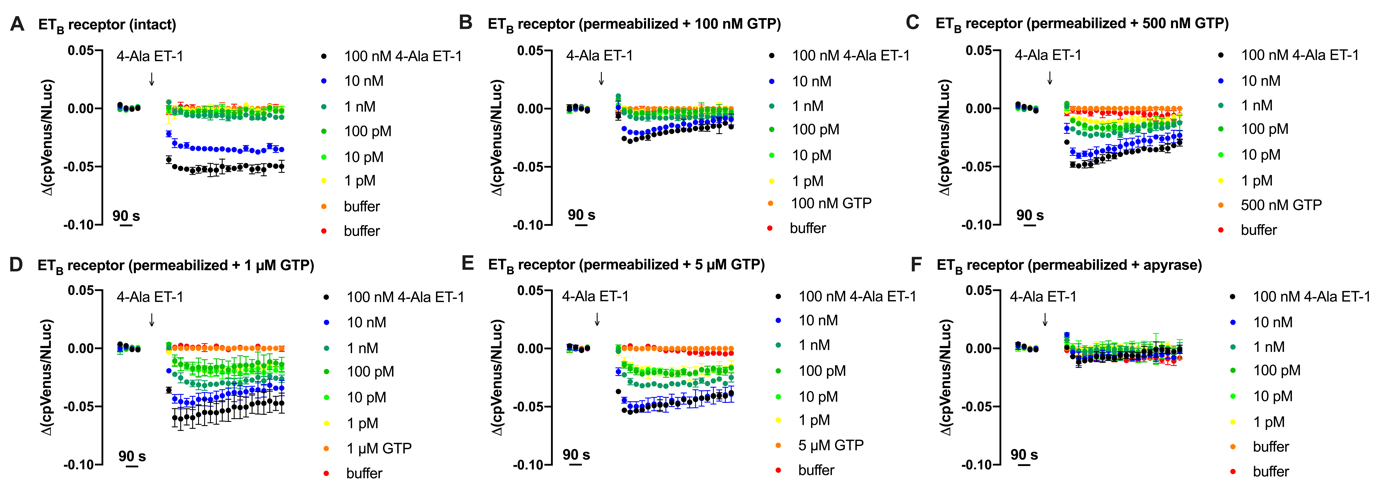


**Supp. Figure 6. Optimization of the GTP concentration for BRET-based G protein activation assay in permeabilized cells.**

(A-F) ΔcpVenus/NLuc time courses of transiently co-transfected HEK293T cells expressing the ET_B_ receptor and BRET-based Gα_q_ protein biosensor (arrow indicates time point of ligand application), (A) in intact cells, (B-E) saponin-permeabilized cells in the presence of 100 nM, 500 nM, 1 µM or 5 µM GTP, respectively, or (F) 2 U/mL apyrase. Data show mean ± SD of three independent experiments.


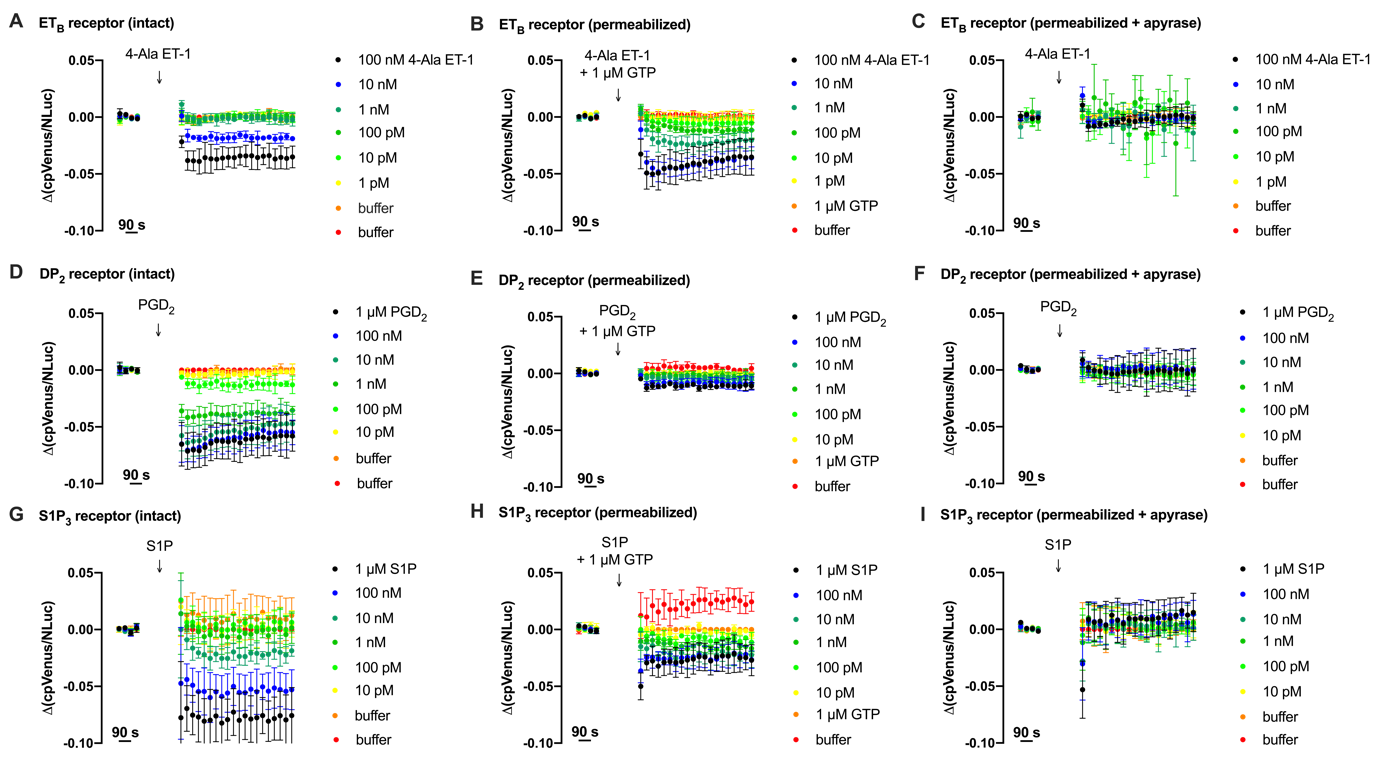


**Supp. Figure 7. BRET-based G protein activation measurements of the ET_B_ receptor, DP_2_ receptor or S1P_3_ receptor in intact or non-intact cells.**

(A-I) ΔcpVenus/NLuc time courses of transiently co-transfected HEK293T cells expressing the (A-C) ET_B_ receptor with BRET-based Gα_q_ biosensor, (D-F) DP_2_ receptor with Gα_o_ biosensor or (G-I) S1P_3_ receptor with Gα_q_ biosensor (arrow indicates time point of ligand application), (A, D, G) in intact cells, (B, E, H) saponin-permeabilized cells in the presence of 1 µM GTP or (C, F, I) 2 U/mL apyrase. The corresponding concentration-response curves are shown in Fig. 4F-H. Data show mean ± SD of three independent experiments.


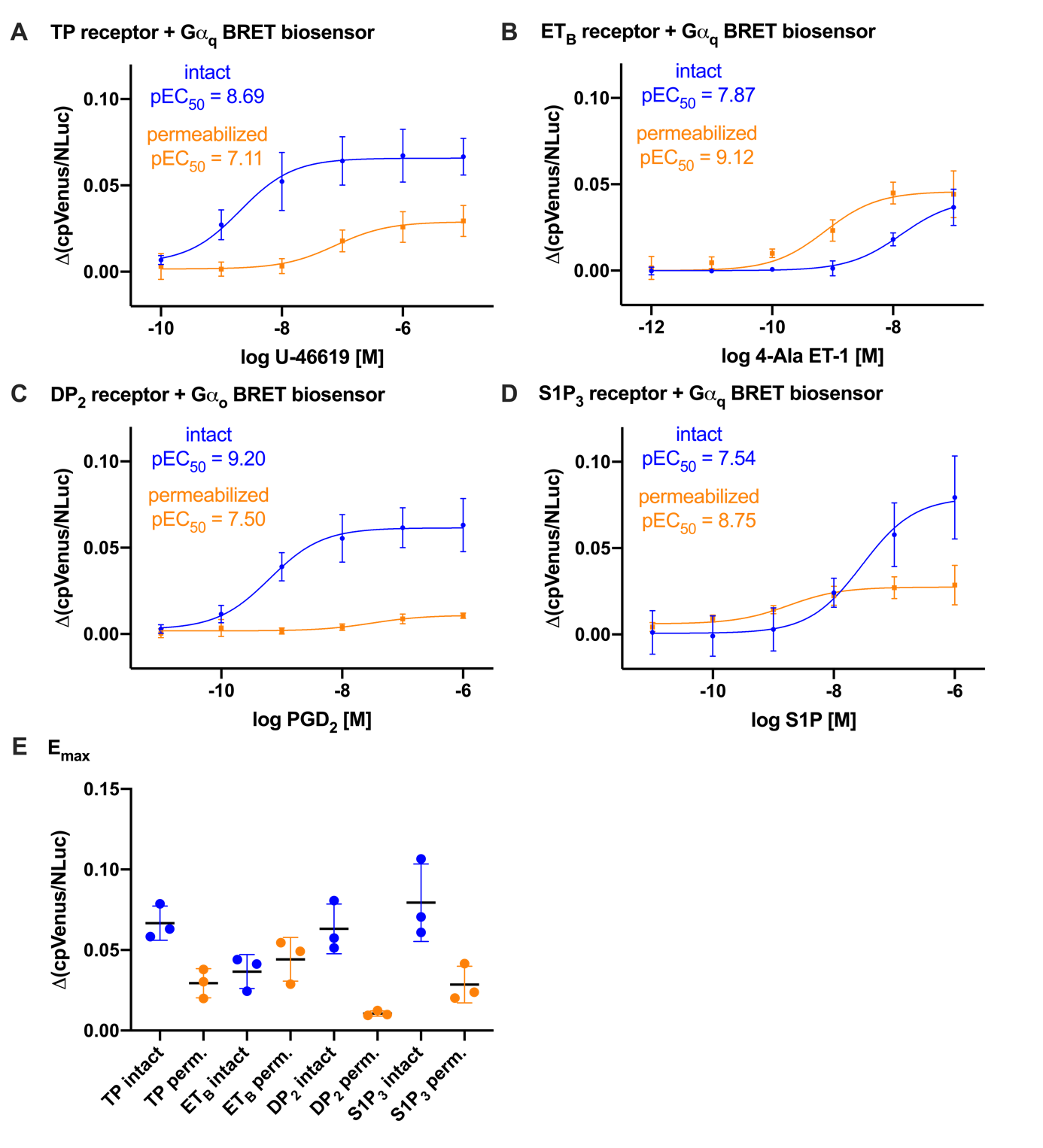


**Supp. Figure 8. Concentration-response curves of BRET-based G protein activation measurements of the TP receptor, ET_B_ receptor, DP_2_ receptor and S1P_3_ receptor without normalization in intact or saponin-permeabilized cells.**

(A-D) Corresponding concentration-response curves of transiently co-transfected HEK293T cells expressing the (A) TP receptor, (B) ET_B_ receptor, (C) DP_2_ receptor or (D) S1P_3_ receptor and BRET-based G protein biosensor. The TP receptor, ET_B_ receptor and S1P_3_ receptor were measured using the Gα_q_ biosensor, while the DP_2_ receptor was measured with the Gα_o_ biosensor. Intact cell measurements are shown in blue and permeabilized cells in orange. (E) shows E_max_ values. The corresponding normalized concentration-response curves are shown in Fig. 4E-H. Data are shown as mean ± SD of three independent experiments.


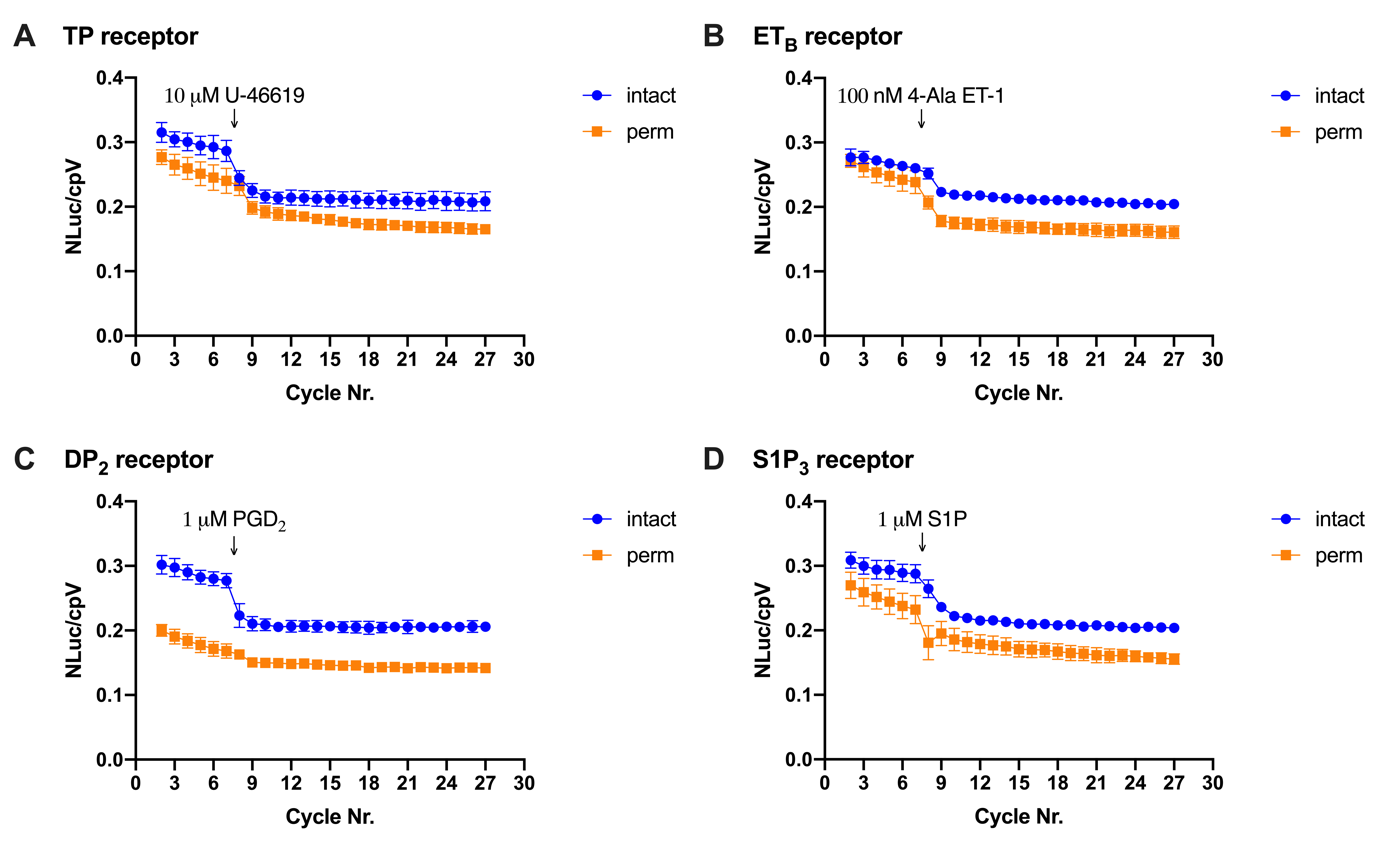


**Supp. Figure 9. BRET-based G protein activation measurements of the TP receptor, ET_B_ receptor, DP_2_ receptor and S1P_3_ receptor without normalization and baseline subtraction in intact or saponin-permeabilized cells.**

(A-D) Measurements of transiently co-transfected HEK293T cells expressing the (A) TP receptor, (B) ET_B_ receptor, (C) DP_2_ receptor or (D) S1P_3_ receptor and BRET-based G protein biosensor. The TP receptor, ET_B_ receptor and S1P_3_ receptor were measured using the Gα_q_ biosensor, while the DP_2_ receptor was measured with the Gα_o_ biosensor. Arrow indicates application of a saturating concentration (E_max_). Intact cell measurements are shown in blue and permeabilized cells in orange. The corresponding normalized concentration-response curves are shown in Fig. 4E-H. Data are shown as mean ± SD of three independent experiments.
